# Convergent-divergent succession of soil microbial communities driven by continuous maize cropping duration via heterogeneous selection processes

**DOI:** 10.3389/fmicb.2025.1618629

**Published:** 2025-06-23

**Authors:** Yan-Liang Han, Fang-Kun Yang, Shu-Ping He, Jia-Cheng Guo, Yue Zou, Yun-Xu Shang, Peng Liu, Peng-Yang Wang, Xing Wang, Ze-Ying Zhao, Juan Wang, Chun-Qing Miao

**Affiliations:** ^1^Zhangye Academy of Agricultural Sciences, Zhangye, China; ^2^Key Laboratory of Biodiversity Formation Mechanism and Comprehensive Utilization of the Qinghai-Tibet Plateau in Qinghai Province, College of Life Sciences, Qinghai Normal University, Xining, China; ^3^State Key Laboratory of Herbage Improvement and Grassland Agro-ecosystems, College of Ecology, Lanzhou University, Lanzhou, China; ^4^Gansu Farms and Land Reclamation Yasheng Group, Gansu Zhongkenyu Seed Industry Co., Ltd., Zhangye, China

**Keywords:** continuous maize cropping, microbial community assembly, convergent-divergent succession, co-occurrence networks, microecological management

## Abstract

The responsive alterations of soil microbial communities driven by continuous maize cropping (CC) and their assembly mechanisms constitute a fundamental scientific question for the sustainability of agricultural ecosystems. However, the assembly processes of microbial communities and their microecological effects under long-term CC remain poorly understood. We hypothesized that maize CC drives predictable shifts in microbial community assembly via increased deterministic selection. To address this, we established a short- to long-term CC gradient (1–25 years). We found that CC significantly altered microbial community structure, the relative abundance of dominant bacterial genera increased with CC years (CCY), whereas dominant fungal genera exhibited a declining trend. Co-occurrence network analysis revealed that long-term CC led to enhanced modularity in bacterial networks and a sharp decline in the proportion of negative correlations. Additionally, our findings reveal a transition from stochastic to heterogeneous selection-dominated processes over time, with microbial diversity metrics showing strong linear relationships with CC duration. This study elucidates how CC shapes convergent-divergent trajectories of microbial communities through heterogeneous selection pressures, providing theoretical support for guiding targeted soil microecological management.

## 1 Introduction

Maize (*Zea mays* L.), a globally vital food and feed crop, is widely cultivated under continuous cropping (CC) systems in intensive agriculture (Zhao Y. et al., 2021). However, long-term CC practices have led to soil degradation and CC-related obstacles, posing critical challenges to agricultural sustainability (Xiong et al., 2022). Studies indicate that community dysbiosis is a central driver of CC-induced constraints (Huang et al., 2023), as it disrupts nutrient cycling, pathogen suppression, and plant-microbe interactions, thereby directly compromising soil ecological functions (Xiong et al., 2022). Additionally, plastic residues accumulated through prolonged film mulching in continuous maize systems may indirectly perturb microbial communities via soil environment modifications (Fu et al., 2023). Although prior research has documented the cumulative effects of CC years (CCY) on microbial diversity (Ma et al., 2023; Zhang et al., 2013), the ecological processes governing microbial succession under CC, particularly community assembly patterns remain poorly resolved. Specifically, the role of deterministic selection pressures (e.g., heterogeneous selection) in mediating long-term adaptive trajectories of microbial communities is yet to be clarified.

Current studies predominantly focus on the direct impacts of CC on microbial community composition (Li G. T. et al., 2023; Han et al., 2019), leaving significant gaps in understanding the ecological mechanisms underlying succession. For instance, microbial communities may transition from stochasticity-dominated to determinism-driven assembly across CC stages, a process tightly coupled with dynamic soil environmental heterogeneity. Notably, community structures may converge under similar selection pressures or weak stochastic processes (Barnett et al., 2019). Conversely, similar initial communities may diverge into distinct structures under environmental filtering from heterogeneous selection pressures (Stegen et al., 2012) (Supplementary Figure S1). Convergent-divergent succession reflects the dynamic trade-off between deterministic and stochastic mechanisms in community assembly (Barnett et al., 2019; Stegen et al., 2012).

Furthermore, existing research largely relies on short-term or discrete temporal comparisons (Huang et al., 2023; Kang et al., 2018; Yang et al., 2025), limiting insights into the convergent-divergent dynamics of communities along continuous CCY gradients. Furthermore, bacteria and fungi, as functionally complementary microbial groups (van der Heijden et al., 2016), may employ distinct strategies to cope with CC-induced stress. Bacteria could maintain metabolic homeostasis through functional redundancy (Ramírez-Puebla et al., 2013), whereas fungal communities are more susceptible to environmental filtering (Kivlin et al., 2014). Therefore, it is necessary to decipher whether the response patterns of these specific taxonomic groups originate from distinct community assembly mechanisms in communities induced by CC.

Additionally, the relationship between the stability of microbial ecosystems and the duration of CC remains unclear. Studies have shown that long-term CC leads to the simplification of soil microbial network structure, increased negative interactions, and reduction of key species (module hubs) to reshape the network (Chen et al., 2018), thereby influencing soil ecosystem resilience and functional robustness (Liu H. et al., 2020; Liu X. et al., 2020). Yet, systematic analyses of network stability evolution along CCY gradients are lacking (Liu H. et al., 2020; Liu X. et al., 2020; Gao D. et al., 2024). This knowledge gap hinders a holistic understanding of CC-driven microecological degradation and the development of microbial-based soil health assessment and management strategies.

This study investigates maize CC systems using a well-defined long-term CC gradient (1–25 years, encompassing seven samples along the gradient) to unravel microbial succession trajectories and their driving mechanisms. The core scientific questions addressed are: (1) how CCY mediates convergent-divergent dynamics of microbial communities through assembly; (2) whether CC enhances the role of deterministic processes in community assembly; (3) whether bacteria and fungi exhibit divergent ecological strategies in response to CC stress; (4) how microbial co-occurrence network topology evolves with CCY and influences microecosystem stability. By integrating community ecology theory with microbiome analytics, this work aims to elucidate the succession patterns and ecological mechanisms of soil microbial communities under CC regimes.

## 2 Materials and methods

### 2.1 Study site and soil sampling

The study was conducted in Zhangye City, Gansu Province, Northwest China (100°27′E, 38°56′N; altitude 1,480 m), a major seed maize production region where long-term CC practices have been implemented due to industrialized planting demands. The area experiences a temperate continental climate characterized by cold, dry winters and warm, arid summers, with an annual mean temperature of 7.5°C, average precipitation of 159 mm, and potential evaporation of 2,200 mm. Soil samples were collected from seed maize fields with seven CCY (1YC, 2YC, 5YC, 10YC, 15YC, 20YC, 25YC). For each CCY, four replicate sampling points were established to ensure data reliability. Thus, this study included a total of seven effective samples (*n* = 7, with 28 subsamples in total). The current CCY gradient was chosen for this study because it reflects the agricultural practice issues in the region, where the majority of the 71,000 ha seed maize fields in Zhangye City have not undergone crop rotation for multiple consecutive years. Specifically, fields with CC exceeding 15 years account for 52.1% (including 9.4% with over 20 years), those with 10–15 years of CC constitute 23.9%, and fields with less than 10 years of CC make up 23.9%. Our study employed a gradient design with 5-year intervals to cover nearly all ranges of CC durations.

Before the CC was carried out, all sampled farmlands adopted a seed corn-potato rotation system. All sampled fields shared identical fertilization and cultivation practices, with consistent climatic conditions across sites due to their proximity. Specifically, all sampling points were within a circular area with a radius of 1.5 km. The sampled plots were either the experimental fields of Zhangye Academy of Agricultural Sciences or the farmlands of farmers guided by the Academy around it. The soil type in the experimental area was all gray-brown desert soil. The drip irrigation volume for seed production corn was 300 mm. The total fertilizer application rate for all seed production corn is 225 kg/ha of N (from urea), 75 kg/ha of P_2_O_5_, and 45 kg/ha of K_2_O. The fertilizers are applied through the drip irrigation system. The corn is planted flat with a density of 72,727 plants/ha, and the ground is covered with plastic film. We collected soil samples (0–20 cm depth) in March 2023 using a soil auger. A five-point sampling method was employed at each site, and subsamples were homogenized and divided into two portions, one air-dried and sieved (2 mm or 0.15 mm stainless steel mesh) for physicochemical analysis, and the other sieved (2 mm mesh) and stored at −80°C for DNA extraction.

### 2.2 Soil physicochemical analysis

Soil organic carbon (SOC), total nitrogen (TN), total phosphorus (TP), total potassium (TK), and pH were analyzed. SOC was determined via the potassium dichromate oxidation-ferrous sulfate titration method. TN was quantified using the semi-micro Kjeldahl method, TP via sulfuric-perchloric acid digestion, and TK by NaOH fusion-flame photometry. Soil pH was measured potentiometrically at a soil-to-water ratio of 1:5 (w/v) (Yang et al., 2025). Residual plastic film content was assessed by collecting topsoil (0–20 cm) from four 1 m × 1 m quadrants per sampling point. Soil was sieved (4 mm mesh), and visible residual film fragments were manually collected, cleaned with an ultrasonic cleaner for 10 min, air-dried, and weighed (Yang et al., 2023).

### 2.3 DNA extraction and sequencing

We extracted total DNA using the TIANGEN DP336 Soil DNA Kit (China). Fungal ITS1 regions were amplified with primers ITS1F (5′-CTTGGTCATTTAGAGGAAGTAA-3′) and ITS2 (5′-GCTGCGTTCTTCATCGATGC-3′), while bacterial 16S rRNA V3–V4 regions were amplified using primers 343F (5′-TACGGRAGGCAGCAG-3′) and 798R (5′-AGGGTATCTAATCCT-3′). PCR conditions included initial denaturation at 94°C for 5 min, followed by 30 cycles of 94°C for 30 s, 55°C for 30 s, and 72°C for 60 s. Amplicons were sequenced on the Illumina NovaSeq6000 platform (United States).

### 2.4 Bioinformatics, co-occurrence network analysis and statistics

Raw sequences were quality-filtered using Trimmomatic (v0.35) and merged with FLASH (v1.2.11). Chimeras were removed via UCHIME (v2.4.2), and high-quality sequences were clustered into operational taxonomic units (OTUs) at 97% similarity using Vsearch (v2.4.2). Taxonomic annotation was performed with the RDP Classifier (v2.13), which assigns taxonomic labels to OTUs by comparing sequences against a reference database with a confidence threshold of 80%. α-diversity indices were calculated using Mothur (v1.30.1). Multivariate analyses (PCoA, PLS-DA, RDA) were conducted in R. Specifically, the vegan package was used for PCoA and RDA, while the ropls package was employed for PLS-DA. Visualization of ordination plots was performed using ggplot2. To investigate the effects of long-term and short-term CC on microbial communities and identify potential biomarkers, LEFSe (Linear discriminant analysis effect size) analysis (LDA threshold = 2, top 150 bacterial and fungal genera) was performed on the Wekemo Bioincloud platform (Gao Y. et al., 2024), while Stamp (Statistical analysis of metagenomic profiles) was performed via the Tutuyun online tool (cloudtutu.com.cn).

For the co-occurrence networks analysis, the “cor.test” function in R was used to construct an adjacency matrix, and a genus-level network analysis was performed for each group of bacteria or fungi. Prior to constructing the adjacency matrix, the data were filtered to retain only those genera with a cumulative relative abundance greater than 0.1%. These networks were composed exclusively of statistically significant and robust correlations (Pearson correlation, *p* < 0.05, |*r*| ≥ 0.7). Here, the Benjamini-Hochberg method (the “p.adjust” function in R) was employed to adjust the *p*-values to reduce the false positive rate of associations. Gephi (v9.2) was utilized for network visualization and the analysis of network topological properties. The robustness of the networks was evaluated using natural connectivity (Wu et al., 2021). Robustness differences between networks were compared by sequentially removing nodes and calculating the percentage of remaining natural connectivity (Wu et al., 2021). Nodes were removed in a targeted manner, starting with those with the highest degree centrality, with one node deleted at a time until 80% of the total nodes were removed. The R code used for robustness analysis is detailed in Supplementary Text S1.

The Neutral Community Model (NCM) was employed to evaluate the potential impacts of stochastic processes on microbial community assembly (Cheng et al., 2024). In this model, the parameter Rsqr (R^2^) serves as an indicator of the overall goodness-of-fit to the neutral model, where Nm represents the metacommunity size multiplied by migration. Fit statistics were calculated using 1,000 iterations of bootstrap with 95% confidence intervals. Genera in each dataset were categorized into three fractions: those with occurrence frequencies higher than (orange), lower than (green), or within the 95% confidence interval predicted by the NCM (neutral fraction, black). The execution of NCM is carried out through the Tutuyun online tool (cloudtutu.com.cn). To characterize the turnover of phylogenetic community composition, we quantified the β-mean nearest taxon distance (βMNTD) (Stegen et al., 2012). The (β-nearest taxon index) βNTI value was used to quantify the deviation of the βMNTD value from the null model distribution (Stegen et al., 2012). Analytical procedures can be found in Zhao W. W. et al. (2021). Statistical analyses (ANOVA, t-tests, LSD *post hoc* tests) were performed using SPSS (v22.0), Excel (2019), and R. Figures were generated with Origin (2018), Excel (2019), and R.

## 3 Results

### 3.1 Effects of continuous maize cropping on soil microbial community structure

**Figure 1:**
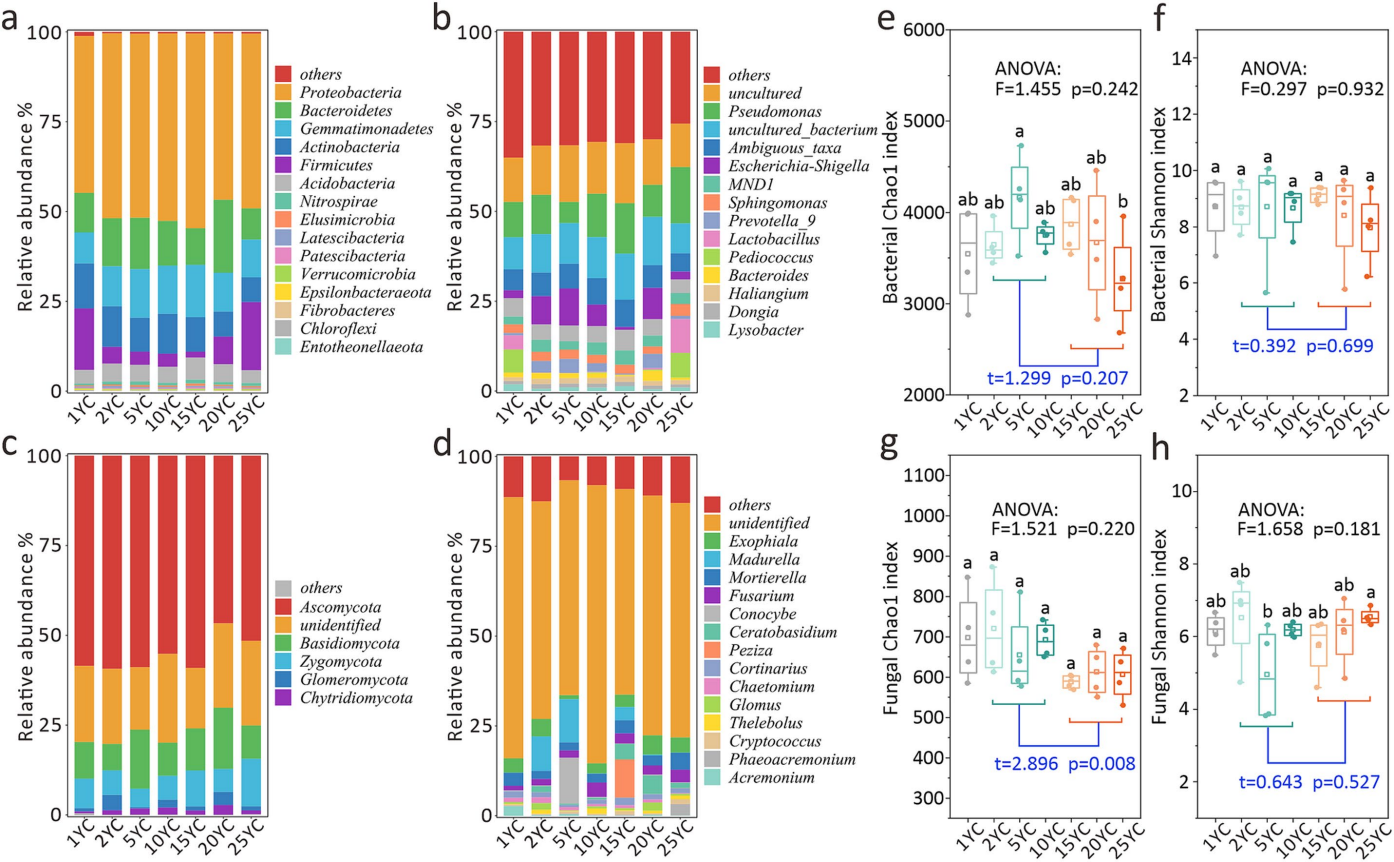
Effects of continuous cropping on soil microbial community structure and diversity. **(a)** Bacterial community structure at phylum level. **(b)** Bacterial community structure at genus level. **(c)** Fungal community structure at phylum level. **(d)** Fungal community structure at genus level. **(e)** Chao1 index of the bacterial community. **(f)** Shannon index of the bacterial community. **(g)** Chao1 index of the fungal community. **(h)** Shannon index of the fungal community. Different letters on the box plot indicate significant differences among the treatments at the 0.05 level (one-way ANOVA). The squares in the boxplot indicate the mean values, while the horizontal lines represent the median values. The *t*-test was used to analyze the differences in indicators between the short-term and long-term CC groups.

CC significantly altered soil microbial community composition (Figure 1; Supplementary Figures S2–S5; Supplementary Tables S1–S4). At the bacterial phylum level, the relative abundances of Gemmatimonadetes (*R*^2^ = 0.700) and Elusimicrobia (*R*^2^ = 0.490) initially increased but later decreased with rising CCY, whereas Firmicutes (*R*^2^ = 0.782) displayed an opposite trend (Figure 1a; Supplementary Figure S2). Actinobacteria abundance in high-CCY groups (20–25YC) was notably lower than that in 1YC (*p* < 0.05), and it exhibited a decreasing trend with the increase of CCY (*R*^2^ = 0.799) (Supplementary Figure S2; Supplementary Table S1). At the genus level, dominant bacterial genera progressively increased in abundance with CCY (*R*^2^ = 0.753) (Supplementary Figure S3). For instance, key genera at 10YC and 25YC exhibited significantly 4.2 and 5.1% higher abundances compared to 1YC, respectively (Figure 1b; Supplementary Table S2). In contrast, fungal communities showed reversed patterns, with dominant fungal genera declining in abundance from 5YC to 25YC (*R*^2^ = 0.653) (Figure 1d; Supplementary Figure S5).

### 3.2 Effects of continuous maize cropping on soil microbial diversity

Bacterial α-diversity analysis revealed minor fluctuations in Chao1 indices across CCY groups (Figure 1e), though inter-group differences were non-significant (ANOVA *p* = 0.242). The 25YC group had marginally lower Chao1 values than other CCY groups, with a significant reduction compared to 5YC (*p* < 0.05) (Figure 1e). Similarly, the bacterial Shannon index showed no significant variations among CCY groups (ANOVA *p* = 0.932) (Figure 1f). While t-tests indicated no significant differences in bacterial α-diversity between short-term (2-10YC) and long-term (15-25YC) CC groups (*p* > 0.05), the Chao1 index was slightly higher in short-term groups (*p* < 0.25). Bacterial Chao1 followed a unimodal pattern, peaking at intermediate CCY (5YC) (Figure 1e).

Fungal α-diversity showed no significant inter-group differences (Chao1 ANOVA *p* = 0.242; Shannon ANOVA *p* = 0.181) (Figures 1g,h). However, the Chao1 index was significantly higher in short-term CC groups than in long-term groups (*p* < 0.01), whereas the Shannon index remained statistically indistinguishable (*p* > 0.05) (Figures 1g,h). Notably, the fungal Shannon index peaked at 25YC and reached its lowest value at 5YC, contrasting with the bacterial Chao1 trend (Figures 1e,h).

**Figure 2:**
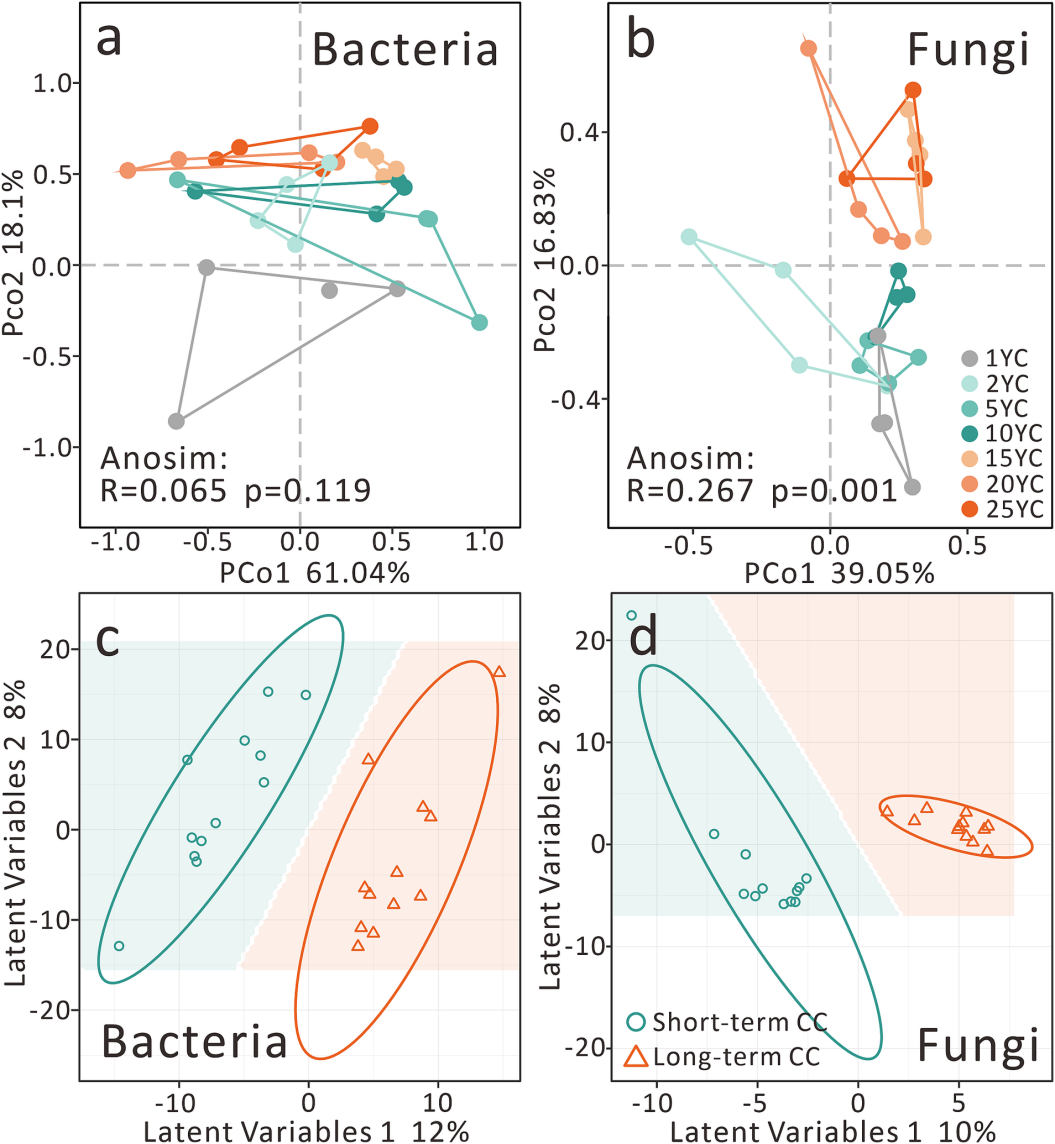
Effects of continuous cropping on soil microbial community β-diversity. **(a)** PCoA of the bacterial community. **(b)** PCoA of the fungal community. **(c)** PLS-DA of the bacterial community. **(d)** PLS-DA of the fungal community. CC, continuous cropping; the same applies hereinafter.

The PCoA of the soil bacterial community showed that the first principal coordinate (PCo1) explained 61.04%, and PCo2 explained 18.1% (Figure 2a). Although there were no significant differences in the bacterial communities among different CCY groups, they still showed considerable grouping stress caused by CCY on PCo2 (Anosim R = 0.065, *p* = 0.119) (Figure 2a). However, we observed that fungal communities exhibited significant differentiation along CCY gradients (PCo1 = 39.05%, PCo2 = 16.83%; Anosim R = 0.267, *p* = 0.001), with distinct clustering along the PCo2 axis (Figure 2b). PLS-DA confirmed significant divergence between short- and long-term CC groups for both bacteria and fungi (Figures 2c,d). Fungal communities demonstrated tighter intra-group clustering, suggesting stronger CCY-driven stress and a higher number of biomarker taxa compared to bacteria.

### 3.3 Analysis of soil microbial biomarkers influenced by continuous maize cropping

**Figure 3:**
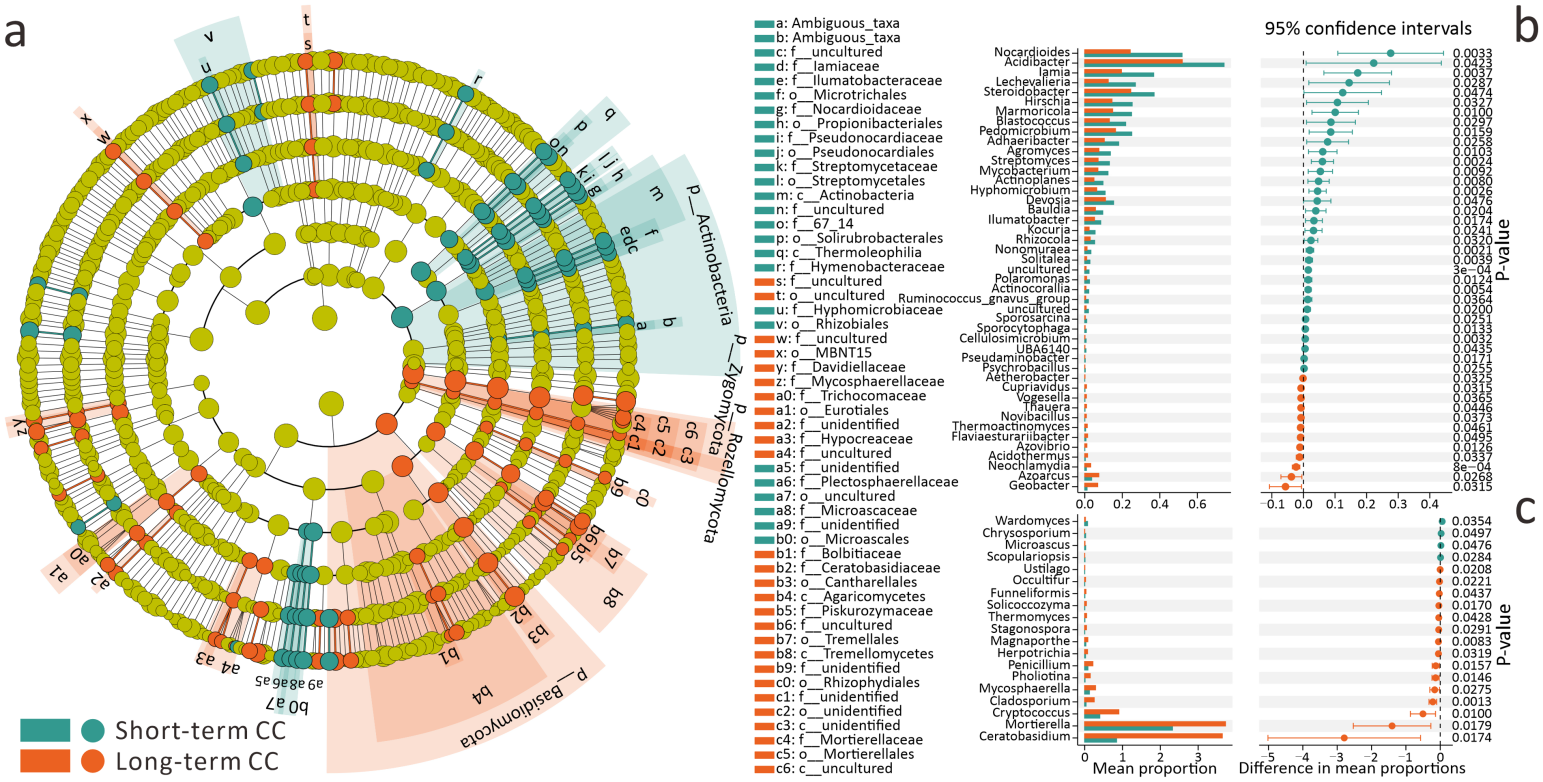
Differential effects of short-term and long-term continuous cropping on soil microbial communities and biomarker analysis. **(a)** LEfSe analysis for identifying bacterial and fungal biomarkers (LDA threshold = 2, top 150 bacterial and fungal genera). **(b)** Stamp analysis for screening bacterial genera-level biomarkers (FDR *q* < 0.05). **(c)** Stamp analysis for screening fungal genera-level biomarkers (FDR *q* < 0.05).

For short-term and long-term CC, using LEfSe analysis, we identified 4 phyla, 6 classes, 16 orders, 29 families, and 45 genera as CC-associated biomarkers (Figure 3a). Fungal biomarkers outnumbered bacterial ones: at the phylum level, Zygomycota, Rozellomycota, and Basidiomycota were fungal biomarkers, while only Actinobacteria represented bacteria. At the genus level, 27 fungal and 18 bacterial biomarkers were identified (Figure 3a). Among these biomarkers, beneficial bacteria (Microtrichales, Nocardioidaceae, Pseudonocardiaceae, Streptomycetales, Streptomycetaceae, Rhizobiales) had higher abundances in the short-term CC group. Conversely, long-term CC significantly increased the abundances of beneficial fungi (Hypocreaceae, Tremellomycetes, Mortierellaceae) (Figure 3a).

It is worth noting that when using Stamp for analysis, due to the inclusion of low-abundance genera and the fact that biomarker selection focuses more on significance rather than effect size, different quantitative trends are presented (Figures 3b,c). Specifically, 45 bacterial and 19 fungal genera were screened as biomarkers. High-abundance bacterial biomarkers were generally more abundant in short-term CC groups (*p* < 0.05), whereas fungal biomarkers predominated in long-term CC groups (*p* < 0.05) (Figures 3b,c). These results align with LEfSe findings, confirming that long-term CC reduces bacterial biomarker abundance but enhances fungal biomarker abundance.

### 3.4 Effects of continuous maize cropping on microbial co-occurrence relationships and stability

**Figure 4:**
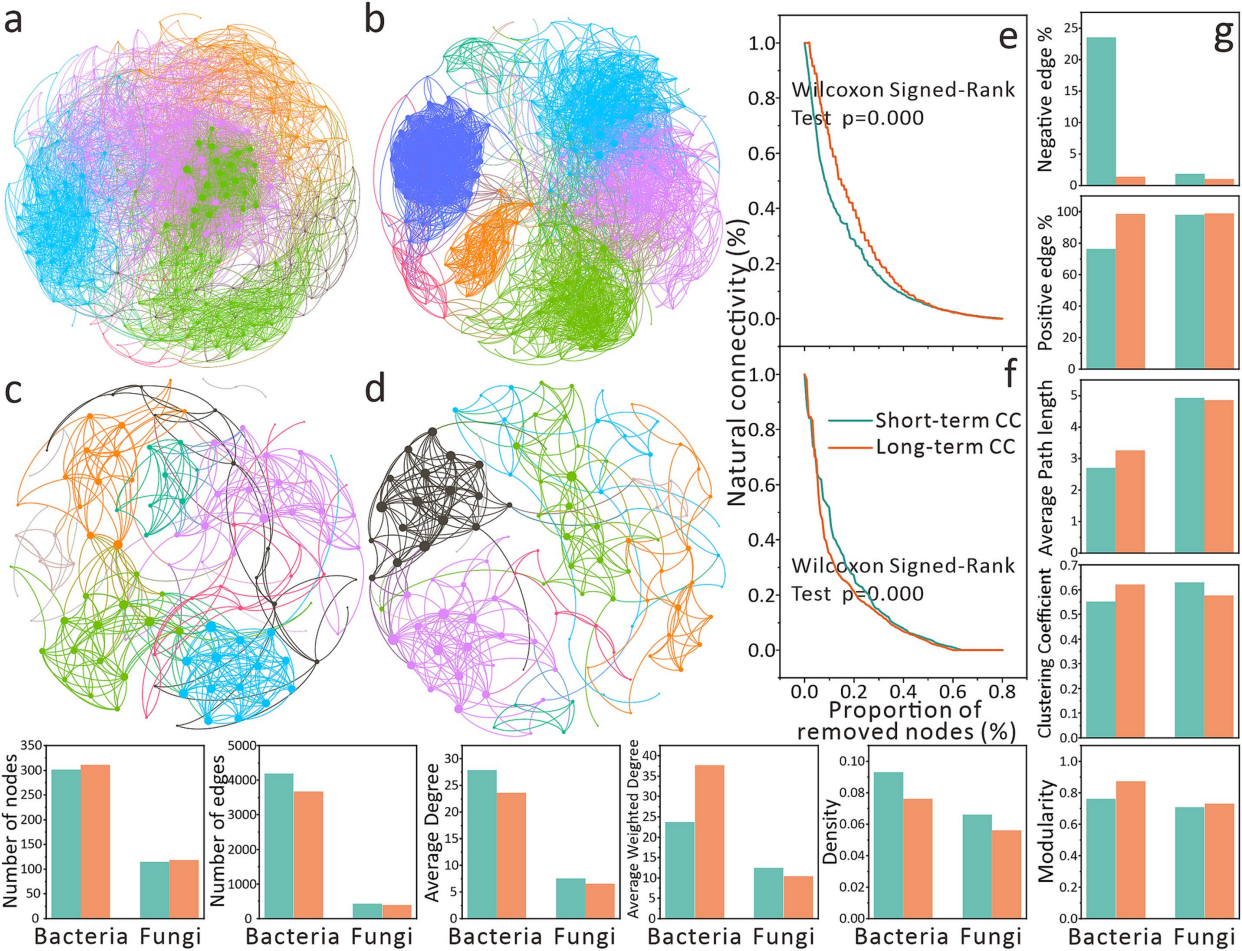
Effects of continuous cropping on co-occurrence relationships of soil microbial communities at the genera level. **(a)** Soil bacterial co-occurrence networks under short-term continuous cropping. **(b)** Soil bacterial co-occurrence networks under long-term continuous cropping. **(c)** Soil fungal co-occurrence networks under short-term continuous cropping. **(d)** Soil fungal co-occurrence networks under long-term continuous cropping. The size of network nodes indicates the degree of node degree, and the same node color indicates that they are in the same module. **(e)** Robustness analysis of bacterial co-occurrence networks. **(f)** Robustness analysis of fungal co-occurrence networks. Network robustness is characterized by natural connectivity. The Wilcoxon Signed-Rank Test was used to analyze the inter-group differences in robustness. **(g)** Topological properties of co-occurrence networks.

The visualization of the network indicates that CC has a differential impact on the co-occurrence relationships and network complexity of the microbial community (Figures 4a–d). Bacterial networks under long-term CC exhibited higher modularity (0.874 vs. 0.762 in short-term CC) (Figures 4a,b,g). Similar but less pronounced trends were observed in fungal networks (Figures 4c,d). While CCY did not affect node numbers, short-term CC bacterial networks contained more edges (4,196 vs. 3,678 in long-term CC).

In both bacterial and fungal networks, short-term CC groups showed higher average degree and density (Figure 4g). Bacterial networks under short-term CC had higher average clustering coefficients and path lengths than long-term CC groups, whereas fungal networks displayed the opposite trend (Figure 4g). All networks exhibited a predominance of positive edges. Notably, short-term CC bacterial networks had a higher proportion of negative edges (23.54% vs. 1.39% in long-term CC), resulting in lower average weighted degrees (Figure 4g).

Subsequently, we evaluated the stability of the co-occurrence network (Figures 4e,f). We found that compared with long-term CC, short-term CC bacterial networks were less stable (*p* < 0.001), with natural connectivity declining rapidly initially but slowing thereafter (Figure 4e). However, long-term CC bacterial networks exhibited faster connectivity loss after 10% node removal (Figure 4e). Fungal networks under short-term CC demonstrated significantly higher stability than that of the long-term CC (*p* = 0.000), and the rate of decrease in natural connectivity of the network showed differences when the nodes were removed by 10–40% (Figure 4f). These findings elucidate the complex CC-driven impacts on microbial co-occurrence ecology (Figure 4).

### 3.5 Response of soil microbial diversity to continuous maize cropping years and predictive potential

**Figure 5:**
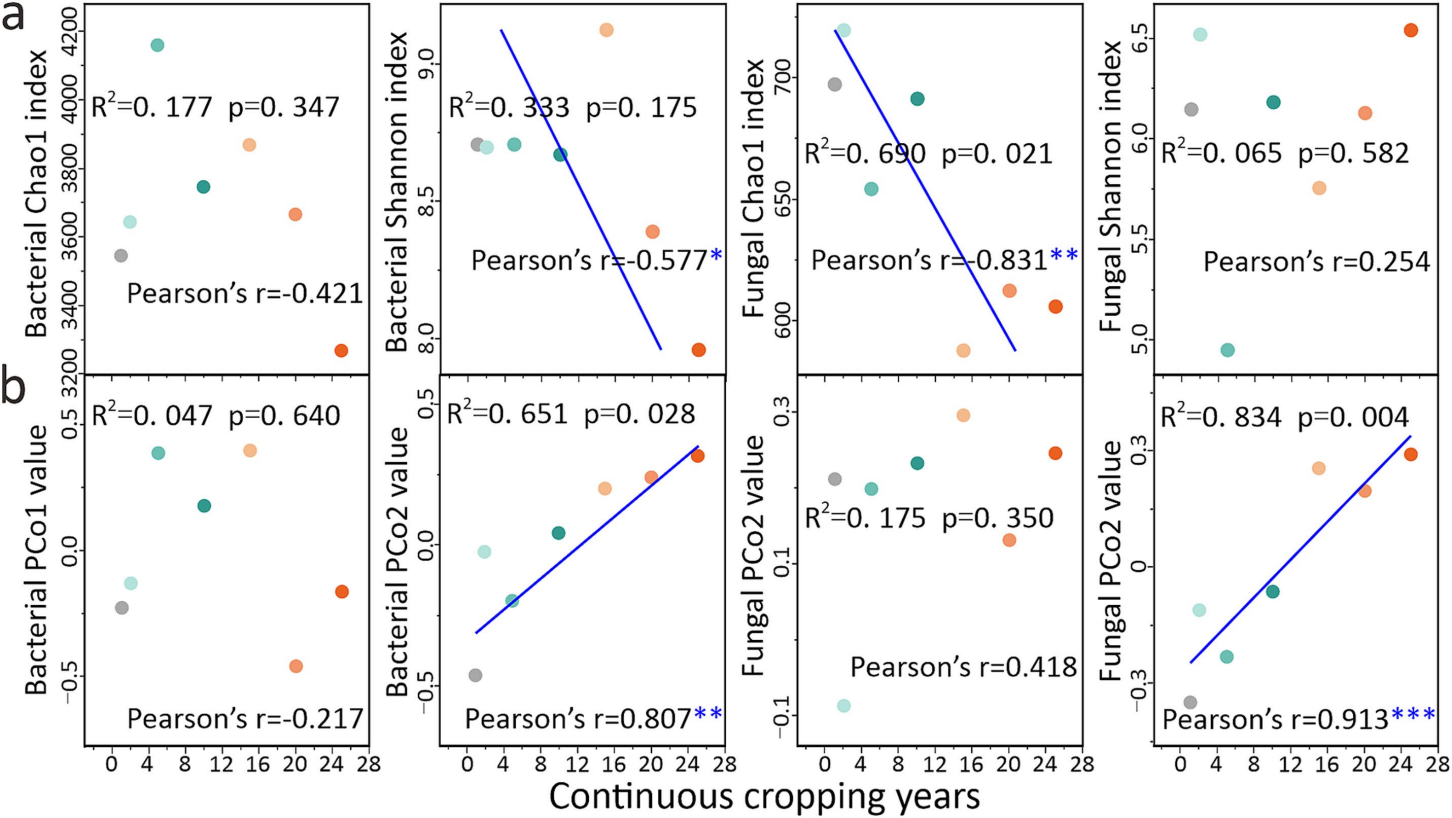
Responses and predictions of soil microbial diversity community characteristics to continuous cropping years. **(a)** Responses and predictions of microbial α-diversity to continuous cropping years (*n* = 7). **(b)** Responses and predictions of microbial β-diversity to continuous cropping years (*n* = 7). Linear regression analysis was conducted, and the *R*^2^ and *p*-values presented in the figure denote the goodness-of-fit and significance level of the linear regression model, respectively. In Pearson correlation analysis, *, **, and *** indicate significant levels of *p* < 0.05, *p* < 0.01, and *p* < 0.001, respectively.

Microbial diversity exhibited a continuous response across the CCY gradient, as revealed by correlation and regression analyses (Figure 5). Specifically, the bacterial Shannon index (Pearson’s *r* = −0.577, *p* < 0.05), fungal Chao1 index (Pearson’s *r* = −0.831, *p* < 0.01), bacterial PCo2 values (Pearson’s *r* = 0.807, *p* < 0.01), and fungal PCo2 values (Pearson’s *r* = 0.913, *p* < 0.001) exhibited significant linear increases or decreases with CCY (Figure 5). Fungal communities displayed stronger CCY-driven responses compared to bacteria. From another perspective, microbial diversity can serve as a good indicator for identifying the CCY of land (Figure 5).

### 3.6 Synergistic effects of continuous maize cropping and soil environmental factors on microbial communities

**Figure 6:**
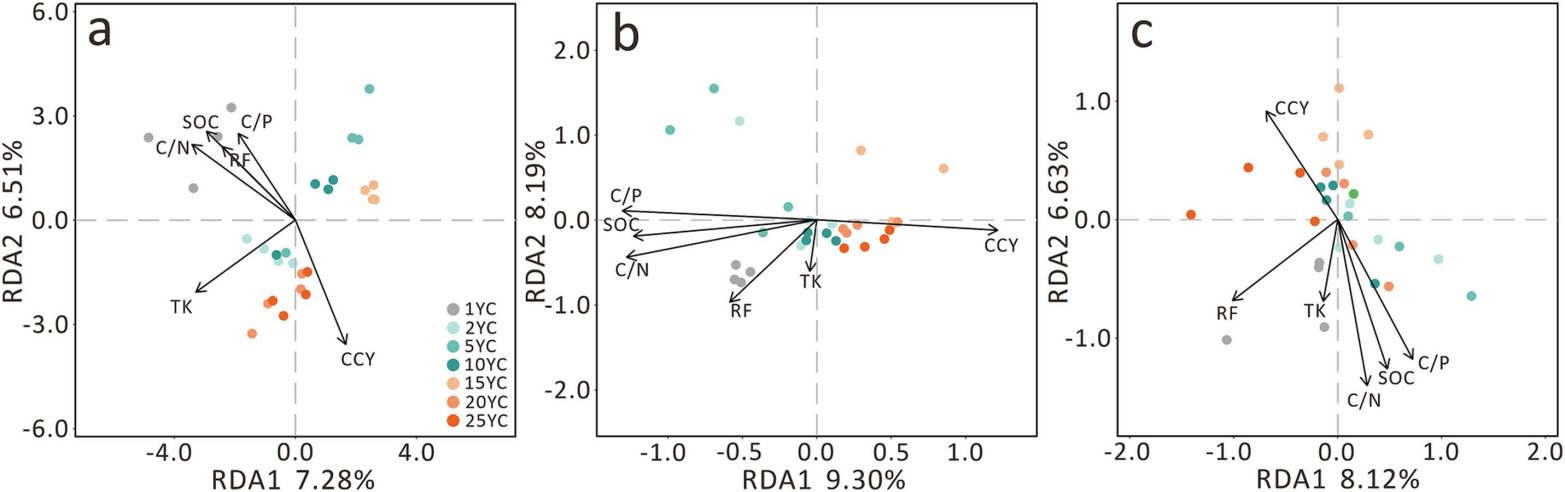
Redundancy analysis (RDA) of the effects of environmental factors and continuous cropping duration on soil microbial community composition. **(a)** Bacterial RDA. **(b)** Fungal RDA. **(c)** Combined Bacterial and Fungal RDA. SOC, soil organic carbon; C/N, carbon to nitrogen ratio; C/P, carbon to phosphorus ratio; TK, total potassium; RF, residual film; CCY, continuous cropping years.

In redundancy analysis (RDA), environmental factors explained 13.79% of bacterial community variation, 17.49% of fungal variation, and 14.75% of combined variation (Figure 6). RDA ordination revealed opposing directional trends between CCY and other environmental variables (Figure 6). Specifically, SOC exhibited a decreasing trend as CCY increased. Similar to β-diversity patterns, distinct clustering of 1YC, short-term CC, and long-term CC groups aligned broadly with CCY vectors, albeit with angular deviations (Figures 2, 6). Notably, TK and residual film (RF) vectors exhibited inconsistent angles across analyses and contributed minimally to RDA1 and RDA2 axes (Figure 6).

### 3.7 Assembly processes of soil microbial communities under continuous maize cropping

**Figure 7:**
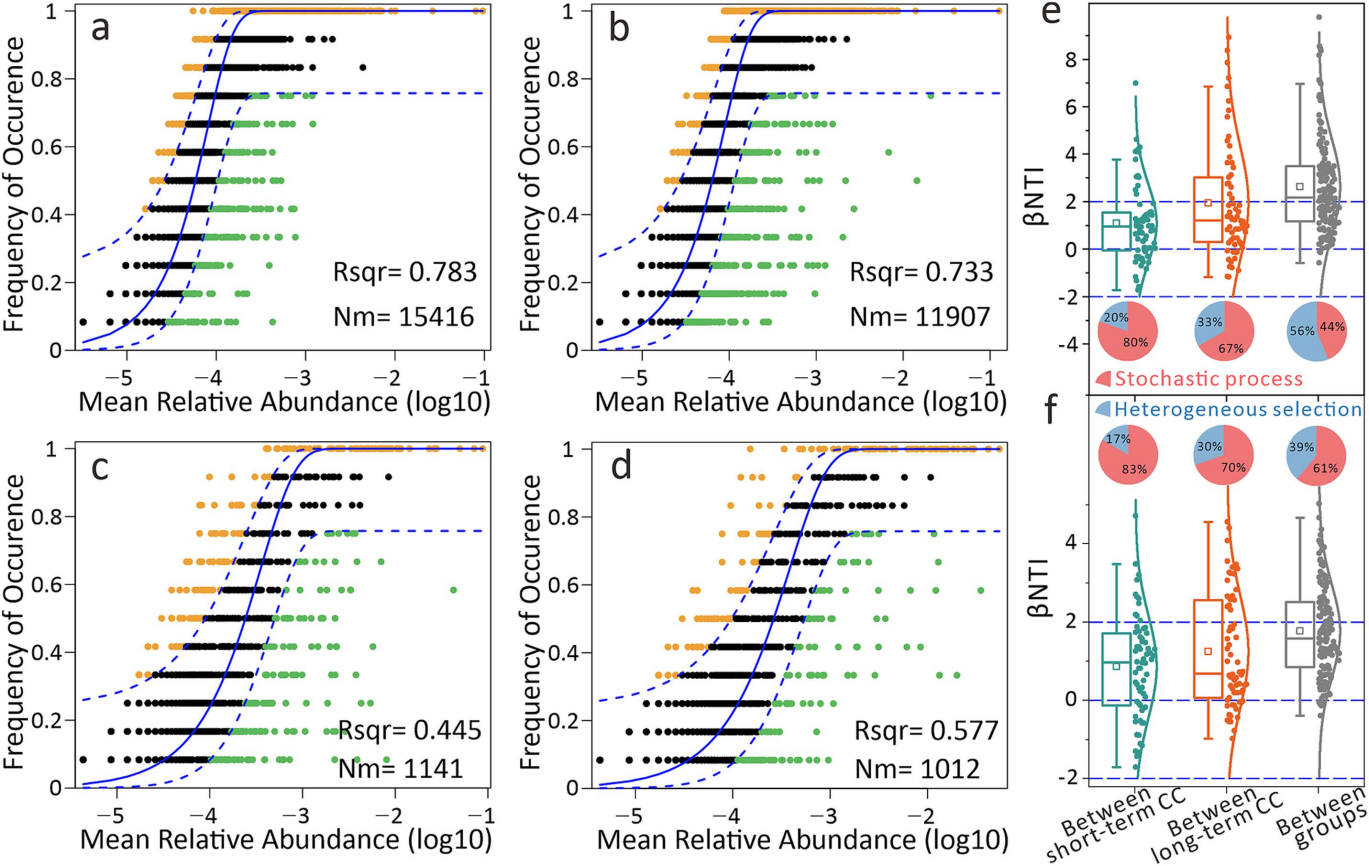
Differential effects of continuous cropping on soil microbial community assembly. **(a)** Neutral community model (NCM) fitting for bacteria under short-term continuous cropping. **(b)** NCM fitting for bacteria under long-term continuous cropping. **(c)** NCM fitting for fungi under short-term continuous cropping. **(d)** NCM fitting for fungi under long-term continuous cropping. Nm represents the metacommunity size multiplied by migration, while Rsqr indicates the model fit. **(e)** Distribution of β-nearest taxon index (βNTI) for the bacterial community. **(f)** βNTI for the fungal community. The squares in the boxplot indicate the mean values, while the horizontal lines represent the median values. The pie chart illustrates the relative proportions of community assembly processes, where the red portion corresponds to stochastic processes and the blue portion reflects deterministic processes, specifically heterogeneous selection.

We applied neutral and null models to dissect community assembly mechanisms. For bacteria, neutral model fitting rates were high in both short-term (78.3%) and long-term CC groups (73.3%) (Figures 7a,b). Fungal communities showed lower but still substantial fitting rates (> 44.5%), indicating stochastic processes dominated assembly (Figures 7c,d). Null model results corroborated these findings, quantifying deterministic vs. stochastic contributions. Heterogeneous selection accounted for 30–33% of assembly in long-term CC groups, a proportion higher than that in short-term groups (17–20%) (Figures 7e,f). Importantly, deterministic processes (heterogeneous selection, 56% for bacteria, 39% for fungi) drove microbial divergence between short- and long-term CC groups, indicating that the changes in the microbial community between the groups were more influenced by deterministic processes.

## 4 Discussion

### 4.1 Divergence of soil microbial communities under deterministic selection pressures

In the context of β-diversity analyses, both bacterial and fungal communities demonstrated notable differentiation along the gradients of CCY (Figure 2). However, fungal communities exhibited a more pronounced divergence, a phenomenon likely attributed to the microenvironmental alterations induced by CC. These alterations encompass a range of factors, including nutrient imbalances that disrupt the natural supply of essential elements for microbial growth (Supplementary Table S5; Supplementary Figure S6), the accumulation of secondary metabolites that may act as antimicrobial agents, the enrichment of plant pathogens that create hostile conditions, and the selection mediated by host root exudates, which can favor certain microbial taxa while inhibiting others (Li et al., 2025; Pervaiz et al., 2020; Ryszkowski et al., 1998). Collectively, these factors impose heterogeneous selection pressures that force microbial communities to adapt over time. Under prolonged stress, such adaptive responses manifest as shifts in the composition of dominant taxa, as documented in previous studies (Li et al., 2025; Pervaiz et al., 2020; Ryszkowski et al., 1998).

A compelling example of such taxonomic shifts is observed in the phylum Actinobacteria, a dominant group highly sensitive to environmental changes. As shown in Figure 1a, Actinobacteria serve as a reliable indicator of CCY, with their abundance showing a marked decline under high CCY conditions. This observation is consistent with findings from European cropping systems, including a 12-year field trial in France that documented Actinobacteria reductions under CC (Romdhane et al., 2022). Additionally, parallel studies in Chinese potato and chrysanthemum CC systems with 8–12 years further corroborate the consistent response of this phylum to prolonged cropping stress (Li J. et al., 2023; Wang et al., 2025).

The observed phenomenon may result either from continuous cropping obstacles induced by the unidirectional accumulation of allelopathic substances (Zhang et al., 2013), or from elevated soil pH levels (Gao et al., 2021). In contrast, crop rotation systems have demonstrated the capacity to preserve soil health through maintaining dynamic equilibrium in allelochemical accumulation and suppressing pathogenic microorganisms (Lu et al., 2024; Dou et al., 2025). In this study, long-term CC significantly reduced the abundance of beneficial bacterial taxa in the soil, such as Microtrichales, Nocardioidaceae, Pseudonocardiaceae, and Rhizobiales, which contribute to soil fertility through carbon-nitrogen cycling, pollutant degradation, or nitrogen fixation, as well as Streptomycetaceae, known for producing streptomycin to suppress pathogens. Conversely, it increased the abundance of beneficial fungal taxa, including Tremellomycetes contributing to biogeochemical processes, Mortierellaceae engaged in plant growth promotion, and Hypocreaceae containing Trichoderma that can serve as biocontrol agents to induce plant resistance (Figure 3). The observed fungal proliferation may be associated with intrinsic pH adaptability, activation of biocontrol mechanisms, K-strategy traits, and symbiotic dependencies in continuously cropped plants. However, the underlying mechanisms require further elucidation through controlled experimental investigations.

Additionally, a paradoxical pattern emerges in bacterial communities, while the overall abundance of dominant bacterial genera increases with CCY, rare taxa experience a decline (Figure 1b). This decline in rare taxa raises concerns about potential impairments to soil functionality, as highlighted by Li Y. et al. (2023). In contrast, dominant fungal taxa exhibit a decrease in abundance with increasing CCY (Figure 1d), reflecting divergent adaptive strategies between bacteria and fungi in response to ecological disturbances (Wang et al., 2023a).

When examining α-diversity, the responses varied significantly across different indices. The bacterial Shannon index and the fungal Chao1 index showed significant correlations with CCY (Figures 5a,b). In contrast, the bacterial Chao1 index and fungal Shannon index did not exhibit such correlations. This discrepancy can be attributed to the fundamental differences between these indices, Chao1 emphasizes species richness, while the Shannon index accounts for both richness and the evenness of species distribution (Finn, 2024; Whittaker, 1972). Our results indicate that CCY primarily reduces bacterial evenness—likely due to the dominance of a few taxa at the expense of others, but there is no significant extinction of rare bacterial species. Bacteria, endowed with rapid metabolic rates, functional redundancy that allows multiple taxa to perform similar functions (Louca et al., 2018), and high adaptability (Cao et al., 2023), tend to adjust their relative abundances (evenness) when coping with CCY-driven stress (Figures 1e,f). In the case of fungi, the decline in richness is a result of the loss of sensitive species without extreme shifts in dominance, thereby preserving evenness despite increasing CCY (Figures 3, 5). These patterns are consistent with findings from a 26-year maize CC study (Liu H. et al., 2020; Liu X. et al., 2020).

### 4.2 Convergence of soil microbial communities under deterministic selection

Neutral and null model analyses revealed that stochastic processes play a dominant role in microbial community assembly, particularly in short-term CC systems (Figure 7). However, as cropping duration extends, long-term CC significantly amplifies the influence of deterministic processes, with heterogeneous selection contributing 30–33% to community assembly. This deterministic force explains the intra-group convergence observed in both bacterial and fungal communities (Figure 2), where environmental constraints such as soil pH, nutrient availability, and stoichiometric ratios act as filters, shaping the composition of microbial taxa (Yang et al., 2024; Rao et al., 2024). These environmental factors drive distinct clustering between short-term (2-10YC) and long-term (15-25YC) CC groups (Figures 2c,d, 3). In global monoculture cropping systems, similar nutrient-driven pressures are evident. Compared to crop rotation, CC systems in the midwestern United States require higher nitrogen fertilizer inputs, exacerbating soil nutrient ratio imbalances (Helmers et al., 2012). Parallel evidence comes from a study conducted in South Africa has shown that CC of legumes under sandy soil conditions leads to enhanced leaching of nutrients such as mineralizable nitrogen (Jani et al., 2022), creating low-nutrient stress that may drive microbial communities to converge toward adapted taxa. Notably, communities within the same group—for example, all long-term CC samples—exhibit higher compositional similarity, underscoring the convergence effect of prolonged environmental filtering. This convergence suggests that over time, the microbial community becomes increasingly shaped by the consistent selection pressures imposed by CC, leading to a reduction in within-group variability and a greater reliance on taxa adapted to the specific stressors of the cropping system.

### 4.3 Trade-offs between co-occurrence network stability and community functionality

Long-term CC enhanced bacterial network modularity but drastically reduced negative correlations (1.39% vs. 23.54% in short-term CC) (Figure 4), suggesting weakened competition and strengthened cooperation—a potential adaptation to resource scarcity or stress (Hernandez et al., 2021). However, diminished competition risks ecological instability. The resulting reduced niche differentiation may enable dominant taxa to monopolize resources (Mougi and Kondoh, 2012; Melián et al., 2009), eroding functional redundancy (Hernandez et al., 2021). While cooperative networks optimize resource use in stable conditions, they exhibit poor resilience to disturbances (e.g., extreme temperatures). Under long-term CC, if the abundance of microbial taxa within mutualistic cooperative frameworks decreases due to other types of stressors, this may trigger structural degradation of cooperative networks via cascading effects. This could ultimately undermine ecosystem stability through the collapse of these critical frameworks (Coyte et al., 2015; Herren and McMahon, 2017; Danczak et al., 2018). Additionally, complex ecosystems like soil rely on modular structures and limited strong interactions for stability (May, 1972). Highly connected modules, though efficient, propagate disruptions rapidly if hub species are compromised (May, 1972; Hernandez et al., 2021).

However, in a study on 25-year CC of tobacco, it was observed that the proportion of negative edges in microbial networks increased due to CC (Chen et al., 2018), which is inconsistent with this study (Figure 4). This discrepancy might be attributed to the fact that tobacco roots release more autotoxic allelochemicals into the soil, and their cumulative effects lead to localized extreme changes in the soil environment, potentially inducing more specialized species and specific competitive interactions among soil microorganisms (Chen et al., 2018; Malard et al., 2022). In contrast, for the maize field soil, as previously mentioned, microorganisms likely cooperate to tackle conventional environmental stresses caused by high-intensity CC (Peng et al., 2024; Hernandez et al., 2021).

### 4.4 Synergistic succession of bacteria and fungi in continuous cropping systems and its soil microecological implications

Despite taxonomic divergences, bacterial and fungal β-diversity shifted synchronously along CCY gradients (Figure 2), reflecting broad-spectrum environmental filtering rather than taxon-specific responses. This synchronized succession suggests microbial communities adapt holistically to CC stress by rebalancing trophic roles (e.g., bacterial decomposers, fungal symbionts/pathogens) to maintain transient ecosystem equilibrium (Teramoto, 1993; Ginzburg and Akçakaya, 1992). However, declining network stability and inverse biomarker trends under long-term CC (Figures 3, 4) signal escalating risks to soil microecosystem integrity. Persistent CC may erode microbial functional resilience, ultimately undermining agricultural sustainability.

### 4.5 Predictive potential of soil microbial community characteristics for continuous cropping duration

The observed divergence-convergence dynamics position soil microbial communities as robust biomarkers for CCY estimation. Prior studies in soybean CC systems linked bacterial community shifts to short-term (7YC) vs. long-term (36YC) differentiation via CPCoA (Yuan et al., 2021), yet sparse CCY gradients limited predictive resolution. Similarly, cotton CC studies reported bacterial divergence between short-term (1–8YC) and long-term (15–20YC) groups, with transitional overlap at 10YC (Ma et al., 2023)—a pattern aligning with our findings. Conversely, greenhouse-based CC systems exhibit abrupt divergence even at minor CCY differences (1–3YC) (Wang et al., 2023b), likely due to reduced soil buffering capacity under intensive management (Wang et al., 2024; Tammaru et al., 2021). Collectively, these studies highlight the need for long-term, continuous CCY datasets to refine microbial-based predictive frameworks.

Based on the findings of this study, we propose a prediction strategy for CCY. For plots with unknown CCY status, if there are adjacent plots with the same crop and known CCY, the CCY threshold of the unknown plot can be estimated by comparing the soil microbial community structures between multiple plots. Moreover, future research could attempt to predict the termination timing of continuous cropping, which would help in formulating more targeted crop rotation strategies. Systematic long-term monitoring of the correlations between CC duration and crop yield, soil pathogen accumulation, as well as the long-term evolution trends of multidimensional soil physicochemical properties and ecological functions, remains necessary in future research. Additionally, expanding the geographic scope of sampling, constructing finer gradient intervals for CC duration, and enhancing the scale of effective sample size are also essential research directions.

## 5 Conclusion

This study elucidates the ecological mechanism whereby long-term maize CC in northwest China’s Gansu province drives convergent-divergent succession in soil microbial communities through heterogeneous selection processes. The results demonstrated that prolonged CCY progressively increased the relative abundance of dominant bacterial genera while significantly reducing fungal richness, with fungal communities exhibiting markedly higher sensitivity to CC stress than their bacterial counterparts. Prolonged CC practices (>15 years) amplified environmental heterogeneous selection pressures, promoting structural convergence within microbial groups and intensifying divergence between groups. This process precipitated a sharp decline in negative correlations within microbial co-occurrence networks, substantially compromising system stability. Notably, microbial diversity metrics showed significant linear correlations with CCY. The findings reveal the adaptive evolutionary dynamics of microbial communities under CC systems in Gansu and their microecological consequences, providing a theoretical framework for soil health assessment in China’s northwestern dryland maize cropping systems. This work advances our understanding of microbial community responses to agricultural intensification while offering empirical evidence for optimizing cropping practices to maintain soil ecosystem functionality.

## Data Availability

The datasets presented in this study can be found in online repositories. The names of the repository/repositories and accession number(s) can be found at: https://www.cncb.ac.cn/, PRJCA040511 (Bacterial GSA number: CRA025951; Fungal GAS number: CRA025953).

## References

[ref1] Barnett S. E. Youngblut N. D. Buckley D. H. (2019). Soil characteristics and land-use drive bacterial community assembly patterns. FEMS Microbiol. Ecol. 96:fiz194. doi: 10.1093/femsec/fiz194, PMID: 31834372

[ref2] Cao H. Li S. He H. Sun Y. Wu Y. Huang Q.. (2023). Stronger linkage of diversity-carbon decomposition for rare rather than abundant bacteria in woodland soils. Front. Microbiol. 14:1115300. doi: 10.3389/fmicb.2023.1115300, PMID: 36937304 PMC10017465

[ref3] Chen S. Qi G. F. Luo T. Zhang H. C. Jiang Q. K. Wang R.. (2018). Continuous-cropping tobacco caused variance of chemical properties and structure of bacterial network in soils. Land Degrad. Dev. 29, 4106–4120. doi: 10.1002/ldr.3167

[ref5] Cheng M. Luo S. Zhang P. Xiong G. Chen K. Jiang C.. (2024). A genome and gene catalog of the aquatic microbiomes of the Tibetan plateau. Nat. Commun. 15:1438. doi: 10.1038/s41467-024-45895-8, PMID: 38365793 PMC10873407

[ref6] Coyte K. Z. Schluter J. Foster K. R. (2015). The ecology of the microbiome: networks, competition, and stability. Science 350, 663–666. doi: 10.1126/science.aad2602, PMID: 26542567

[ref7] Danczak R. E. Johnston M. D. Kenah C. Slattery M. Wilkins M. J. (2018). Microbial community cohesion mediates community turnover in unperturbed aquifers. mSystems 3:e00066-18. doi: 10.1128/mSystems.00066-18 29984314 PMC6030547

[ref8] Dou Y. Yu S. Liu S. Cui T. Huang R. Wang Y.. (2025). Crop rotations reduce pathogenic fungi compared to continuous cropping. Rhizosphere 34:101074. doi: 10.1016/j.rhisph.2025.101074

[ref9] Finn D. R. (2024). A metagenomic alpha-diversity index for microbial functional biodiversity. FEMS Microbiol. Ecol. 100:fiae019. doi: 10.1093/femsec/fiae019, PMID: 38337180 PMC10939414

[ref10] Fu F. Long B. Huang Q. Li J. Zhou W. Yang C. (2023). Integrated effects of residual plastic films on soil-rhizosphere microbe-plant ecosystem. J. Hazard. Mater. 445:130420. doi: 10.1016/j.jhazmat.2022.130420, PMID: 36462237

[ref11] Gao D. Gao X. S. Wang Y. Huo H. M. Wu Y. H. Yang Z. M.. (2024). Effects of long-term continuous cultivation on the structure and function of soil bacterial and fungal communities of *Fritillaria cirrhosa* on the Qinghai-Tibetan plateau. Sci. Rep. 14:21291. doi: 10.1038/s41598-024-70625-x 39266574 PMC11393089

[ref12] Gao Z. Hu Y. Han M. Xu J. Wang X. Liu L.. (2021). Effects of continuous cropping of sweet potatoes on the bacterial community structure in rhizospheric soil. BMC Microbiol. 21:102. doi: 10.1186/s12866-021-02120-6, PMID: 33794774 PMC8015022

[ref13] Gao Y. Zhang G. Jiang S. Liu Y. X. (2024). Wekemo Bioincloud: a user-friendly platform for meta-omics data analyses. iMeta 3:e175. doi: 10.1002/imt2.175, PMID: 38868508 PMC10989175

[ref14] Ginzburg L. R. Akçakaya H. R. (1992). Consequences of ratio-dependent predation for steady-state properties of ecosystems. Ecology 73, 1536–1543. doi: 10.2307/1940006

[ref15] Han G. M. Chen Q. Q. Zhang S. X. Li G. R. Yi X. D. Feng C. H.. (2019). Biochar effects on bacterial community and metabolic pathways in continuously cotton - cropped soil. J. Soil Sci. Plant Nutr. 19, 249–261. doi: 10.1007/s42729-019-0014-z

[ref16] Helmers M. J. Zhou X. Baker J. L. Melvin S. W. Lemke D. W. (2012). Nitrogen loss on tile-drained Mollisols as affected by nitrogen application rate under continuous corn and corn-soybean rotation systems. Can. J. Soil Sci. 92, 493–499. doi: 10.4141/cjss2010-043

[ref17] Hernandez D. J. David A. S. Menges E. S. Searcy C. A. Afkhami M. E. (2021). Environmental stress destabilizes microbial networks. ISME J. 15, 1722–1734. doi: 10.1038/s41396-020-00882-x, PMID: 33452480 PMC8163744

[ref18] Herren C. M. McMahon K. D. (2017). Cohesion: a method for quantifying the connectivity of microbial communities. ISME J. 11, 2426–2438. doi: 10.1038/ismej.2017.91, PMID: 28731477 PMC5649174

[ref19] Huang Z. B. Wang X. Fan L. S. Jin X. J. Zhang X. Wang H. Y. (2023). Continuous cropping of *Tussilago farfara* L. has a significant impact on the yield and quality of its flower buds, and physicochemical properties and the microbial communities of rhizosphere soil. Life 15:404. doi: 10.3390/life15030404 PMC11944208 40141749

[ref20] Jani A. D. Motis T. N. Longfellow J. M. Lingbeek B. J. D’Aiuto C. J. (2022). Continuous cropping legumes in semi-arid southern Africa: legume productivity and soil health implications. Exp. Agric. 58:e15. doi: 10.1017/S0014479722000138

[ref21] Kang Y. L. Jing F. Sun W. Q. Liu J. G. Jiang G. Y. (2018). Soil microbial communities changed with a continuously monocropped processing tomato system. Acta Agricult. Scand. B Soil Plant Sci. 68, 149–160. doi: 10.1080/09064710.2017.1370124

[ref22] Kivlin S. N. Winston G. C. Goulden M. L. Treseder K. K. (2014). Environmental filtering affects soil fungal community composition more than dispersal limitation at regional scales. Fungal Ecol. 12, 14–25. doi: 10.1016/j.funeco.2014.04.004

[ref23] Li J. Cheng X. Chu G. Hu B. Tao R. (2023). Continuous cropping of cut chrysanthemum reduces rhizospheric soil bacterial community diversity and co-occurrence network complexity. Appl. Soil Ecol. 185:104801. doi: 10.1016/j.apsoil.2022.104801

[ref24] Li Y. Gao W. Wang C. Gao M. (2023). Distinct distribution patterns and functional potentials of rare and abundant microorganisms between plastisphere and soils. Sci. Total Environ. 873:162413. doi: 10.1016/j.scitotenv.2023.162413, PMID: 36842601

[ref25] Li G. T. Gong P. F. Zhou J. Wang L. Song X. Ding P. H.. (2023). The succession of rhizosphere microbial community in the continuous cropping soil of tobacco. Front. Environ. Sci. 11:1251938. doi: 10.3389/fenvs.2023.1251938

[ref26] Li J. Xu Z. Yang T. Zhang J. Zuo Y. Cheng L. (2025). Rhizosphere ecological restoration: interactions between nutrient mobilization, core microbial assembly, and phenylalanine metabolism circulation. Biochar 7:64. doi: 10.1007/s42773-024-00402-6

[ref27] Liu X. Li Y. J. Ren X. J. Chen B. H. Zhang Y. Shen C. W.. (2020). Long-term greenhouse cucumber production alters soil bacterial community structure. J. Soil Sci. Plant Nutr. 20, 306–321. doi: 10.1007/s42729-019-00109-9

[ref28] Liu H. Pan F. Han X. Song F. Zhang Z. Yan J.. (2020). A comprehensive analysis of the response of the fungal community structure to long-term continuous cropping in three typical upland crops. J. Integr. Agric. 19, 866–880. doi: 10.1016/S2095-3119(19)62630-4

[ref29] Louca S. Polz M. F. Mazel F. Albright M. B. N. Huber J. A. O'Connor M. I.. (2018). Function and functional redundancy in microbial systems. Nat. Ecol. Evol. 2, 936–943. doi: 10.1038/s41559-018-0519-1, PMID: 29662222

[ref30] Lu J. Liu Y. Zou X. Zhang X. Yu X. Wang Y.. (2024). Rotational strip peanut/cotton intercropping improves agricultural production through modulating plant growth, root exudates, and soil microbial communities. Agric. Ecosyst. Environ. 359:108767. doi: 10.1016/j.agee.2023.108767

[ref31] Ma Z. Li P. Yang C. Feng Z. Feng H. Zhang Y.. (2023). Soil bacterial community response to continuous cropping of cotton. Front. Microbiol. 14:1125564. doi: 10.3389/fmicb.2023.1125564, PMID: 36778850 PMC9909236

[ref33] Malard L. A. Mod H. K. Guex N. Broennimann O. Yashiro E. Lara E.. (2022). Comparative analysis of diversity and environmental niches of soil bacterial, archaeal, fungal and protist communities reveal niche divergences along environmental gradients in the Alps. Soil Biol. Biochem. 169:108674. doi: 10.1016/j.soilbio.2022.108674

[ref34] May R. M. (1972). Will a large complex system be stable? Nature 238, 413–414. doi: 10.1038/238413a0, PMID: 4559589

[ref35] Melián C. J. Bascompte J. Jordano P. Kriván V. (2009). Diversity in a complex ecological network with two interaction types. Oikos 118, 122–130. doi: 10.1111/j.1600-0706.2008.16751.x

[ref36] Mougi A. Kondoh M. (2012). Diversity of interaction types and ecological community stability. Science 337, 349–351. doi: 10.1126/science.1220529, PMID: 22822151

[ref37] Peng Z. Qian X. Liu Y. Li X. Gao H. An Y.. (2024). Land conversion to agriculture induces taxonomic homogenization of soil microbial communities globally. Nat. Commun. 15:3624. doi: 10.1038/s41467-024-47348-8, PMID: 38684659 PMC11058813

[ref38] Pervaiz Z. H. Iqbal J. Zhang Q. Chen D. Wei H. Saleem M. (2020). Continuous cropping alters multiple biotic and abiotic indicators of soil health. Soil Syst. 4:59. doi: 10.3390/soilsystems4040059

[ref39] Ramírez-Puebla S. T. Servín-Garcidueñas L. E. Jiménez-Marín B. Bolaños L. M. Rosenblueth M. Martínez J.. (2013). Gut and root microbiota commonalities. Appl. Environ. Microbiol. 79, 2–9. doi: 10.1128/AEM.02553-12, PMID: 23104406 PMC3536091

[ref40] Rao G. Song W. L. Yan S. Z. Chen S. L. (2024). Unraveling the distribution pattern and driving forces of soil microorganisms under geographic barriers. Appl. Environ. Microbiol. 90, e01359–e01324. doi: 10.1128/aem.01359-24, PMID: 39171904 PMC11409670

[ref41] Romdhane S. Spor A. Banerjee S. Breuil M.-C. Bru D. Chabbi A.. (2022). Land-use intensification differentially affects bacterial, fungal and protist communities and decreases microbiome network complexity. Environ. Microb. 17:1. doi: 10.1186/s40793-021-00396-9, PMID: 34991714 PMC8740439

[ref42] Ryszkowski L. Szajdak L. Karg J. (1998). Effects of continuous cropping of rye on soil biota and biochemistry. Crit. Rev. Plant Sci. 17, 225–244. doi: 10.1080/07352689891304221

[ref43] Stegen J. C. Lin X. Konopka A. E. Fredrickson J. K. (2012). Stochastic and deterministic assembly processes in subsurface microbial communities. ISME J. 6, 1653–1664. doi: 10.1038/ismej.2012.22, PMID: 22456445 PMC3498916

[ref44] Tammaru K. Košnar J. Abbas A. F. Barta K. A. de Bello F. Harrison S.. (2021). Ecological differentiation of *Carex* species coexisting in a wet meadow: comparison of pot and field experiments. Acta Oecol. 110:103692. doi: 10.1016/j.actao.2020.103692

[ref45] Teramoto E. (1993). Dynamical structure of energy trophic levels. Ecol. Model. 69, 135–147. doi: 10.1016/0304-3800(93)90053-U

[ref46] van der Heijden M. G. A. de Bruin S. Luckerhoff L. van Logtestijn R. S. P. Schlaeppi K. (2016). A widespread plant-fungal-bacterial symbiosis promotes plant biodiversity, plant nutrition and seedling recruitment. ISME J. 10, 389–399. doi: 10.1038/ismej.2015.120, PMID: 26172208 PMC4737930

[ref47] Wang Z. Leite M. F. A. Jiang M. Kuramae E. E. Fu X. (2023a). Responses of soil rare and abundant microorganisms to recurring biotic disturbances. Soil Biol. Biochem. 177:108913. doi: 10.1016/j.soilbio.2022.108913

[ref48] Wang J. Li M. Zhou Q. Zhang T. (2023b). Effects of continuous cropping Jiashi muskmelon on rhizosphere microbial community. Front. Microbiol. 13:1086334. doi: 10.3389/fmicb.2022.1086334, PMID: 36699602 PMC9868712

[ref49] Wang L. Liu S. Tian G. Pan Y. Wang H. Qiu G.. (2025). Impacts of continuous potato cropping on soil microbial assembly processes and spread of potato common scab. Appl. Soil Ecol. 206:105805. doi: 10.1016/j.apsoil.2024.105805

[ref50] Wang P. Y. Zhao Z. Y. Siddique K. H. M. Xiong X. B. Tao H. Y. Ma Y.. (2024). Microplastics positively mediate soil multifunctionality in dryland. Resour. Conserv. Recycl. 209:107754. doi: 10.1016/j.resconrec.2024.107754

[ref51] Whittaker R. H. (1972). Evolution and measurement of species diversity. Taxon 21, 213–251. doi: 10.2307/1218190

[ref52] Wu M. H. Chen S. Y. Chen J. W. Xue K. Chen S. L. Wang X. M.. (2021). Reduced microbial stability in the active layer is associated with carbon loss under alpine permafrost degradation. Proc. Natl. Acad. Sci. 118:e2025321118. doi: 10.1073/pnas.2025321118, PMID: 34131077 PMC8237688

[ref53] Xiong J. Peng S. G. Liu Y. J. Yin H. Q. Zhou L. Zhou Z. C.. (2022). Soil properties, rhizosphere bacterial community, and plant performance respond differently to fumigation and bioagent treatment in continuous cropping fields. Front. Microbiol. 13:923405. doi: 10.3389/fmicb.2022.923405, PMID: 35935223 PMC9354655

[ref54] Yang Z. Dai H. Huang Y. Dong B. Fu S. Zhang C.. (2024). Driving mechanisms of soil bacterial α and β diversity under long-term nitrogen addition: subtractive heterogenization based on the environment selection. Geoderma 445:116886. doi: 10.1016/j.geoderma.2024.116886

[ref55] Yang B. Y. Feng C. C. Jiang H. Chen Y. L. Ding M. J. Dai H. X.. (2025). Effects of long-term continuous cropping on microbial community structure and function in tobacco rhizosphere soil. Front. Microbiol. 16:1496385. doi: 10.3389/fmicb.2025.1496385, PMID: 40160271 PMC11949956

[ref56] Yang L. L. Heng T. He X. L. Yang G. Zhao L. Li Y. H.. (2023). Spatial-temporal distribution and accumulation characteristics of residual plastic film in cotton fields in arid oasis area and the effects on soil salt transport and crop growth. Soil Tillage Res. 231:105737. doi: 10.1016/j.still.2023.105737

[ref57] Yuan M. Yu T. Shi Q. Han D. Yu K. Wang L.. (2021). Rhizosphere soil bacterial communities of continuous cropping-tolerant and sensitive soybean genotypes respond differently to long-term continuous cropping in Mollisols. Front. Microbiol. 12:729047. doi: 10.3389/fmicb.2021.729047, PMID: 34589076 PMC8473881

[ref58] Zhang W. Long X. Q. Huo X. D. Chen Y. F. Lou K. (2013). 16S rRNA-based PCR-DGGE analysis of actinomycete communities in fields with continuous cotton cropping in Xinjiang, China. Microb. Ecol. 66, 385–393. doi: 10.1007/s00248-012-0160-5, PMID: 23299346

[ref59] Zhao Y. Fu W. Hu C. Chen G. Xiao Z. Chen Y.. (2021). Variation of rhizosphere microbial community in continuous mono-maize seed production. Sci. Rep. 11:1544. doi: 10.1038/s41598-021-81228-1, PMID: 33452372 PMC7810720

[ref60] Zhao W. W. Wang X. B. Shi L. Y. Zhu W. Y. Ma L. Wang J. J.. (2021). Calculation method for stochastic and deterministic assembly processes of prokaryotic communities. BioProtocol 11:e2003400. doi: 10.21769/BioProtoc.2003400

