# Supplementary material for: Convergent-divergent succession of soil microbial communities driven by continuous maize cropping duration via heterogeneous selection processes
Source: Front Microbiol. 2025 Jun 23;16:1618629. doi: 10.3389/fmicb.2025.1618629 (PMC12230030; doi:10.3389/fmicb.2025.1618629)
Supplement: Supplementary file 1 [file Data_Sheet_1.docx]

**Supporting Information for**

**Convergent-divergent succession of soil microbial communities driven by continuous maize cropping duration via heterogeneous selection processes**

Yan-Liang Han^a^†, Fang-Kun Yang^b,c^†, Shu-Ping He^a^, Jia-Cheng Guo^c^, Yue Zou^a^, Yun-Xu Shang^c^, Peng Liu^d^, Peng-Yang Wang^c^, Xing Wang^a^, Ze-Ying Zhao^c^, Juan Wang^a^*, Chun-Qing Miao^a^*

^a^Zhangye Academy of Agricultural Sciences, Zhangye 734000, China.

^b^Key Laboratory of Biodiversity Formation Mechanism and Comprehensive Utilization of the Qinghai-Tibet Plateau in Qinghai Province, Qinghai Normal University, Xining, 810008, China.

^c^State Key Laboratory of Herbage Improvement and Grassland Agro-ecosystems, College of Ecology, Lanzhou Universit, Lanzhou 730000, China.

^d^Gansu Farms and Land Reclamation Yasheng Group, Gansu Zhongkenyu Seed Industry Co., Ltd., Zhangye 734000, China.

*Corresponding author.

†These authors contributed equally to this work.

Email: miaocq3139@163.com (C.Q. Miao*); [wangjuan_8162@163.com](mailto:wangjuan_8162@163.com) (J. Wang*)

**Table S****1 Analysis of the differences in the relative abundance of dominant species of bacteria at the phylum level**.

|  | 1YC | 2YC | 5YC | 10YC | 15YC | 20YC | 25YC |
| --- | --- | --- | --- | --- | --- | --- | --- |
| Gemmatimonadetes | 0.0861c | 0.1111abc | 0.1352ab | 0.1336ab | 0.1455a | 0.1081abc | 0.1050bc |
| Actinobacteria | 0.1256a | 0.1134ab | 0.0945ab | 0.1114ab | 0.0961ab | 0.0698b | 0.0690b |
| Firmicutes | 0.1709a | 0.0463b | 0.0366b | 0.0364b | 0.0166b | 0.0768b | 0.1896a |
| Nitrospirae | 0.0047b | 0.0079ab | 0.0095ab | 0.0071ab | 0.0103a | 0.0072ab | 0.0083ab |
| Elusimicrobia | 0.0030b | 0.0043ab | 0.0057ab | 0.0046ab | 0.0073a | 0.0046ab | 0.0049ab |
| Latescibacteria | 0.0027b | 0.0055a | 0.0031b | 0.0030b | 0.0036ab | 0.0038ab | 0.0017b |
| Fibrobacteres | 0.0006c | 0.0010bc | 0.0008bc | 0.0010bc | 0.0017a | 0.0008bc | 0.0012b |
| Chloroflexi | 0.0019a | 0.0008b | 0.0010b | 0.0008b | 0.0009b | 0.0007b | 0.0007b |
| others | 0.0110a | 0.0038b | 0.0048b | 0.0041b | 0.0043b | 0.0045b | 0.0047b |

Note: The analysis was conducted among the top 15 phyla with the highest bacterial abundance, and only the phyla with significant differences were presented. The numbers represent the relative abundance of the phyla, and different lowercase letters indicate significant differences (ANOVA LSD p<0.05).

**Table S2 Analysis of the differences in the relative abundance of dominant species of bacteria at the genus level**.

|  | 1YC | 2YC | 5YC | 10YC | 15YC | 20YC | 25YC |
| --- | --- | --- | --- | --- | --- | --- | --- |
| Pseudomonas | 0.0985ab | 0.1096ab | 0.0591b | 0.1211ab | 0.1405ab | 0.0895ab | 0.1568a |
| MND1 | 0.0216b | 0.0333ab | 0.0233ab | 0.0346ab | 0.0396a | 0.0300ab | 0.0302ab |
| Lysobacter | 0.0187a | 0.0064c | 0.0101bc | 0.0095bc | 0.0137ab | 0.0051bc | 0.0094c |
| others | 0.3504a | 0.3167ab | 0.3160ab | 0.3066ab | 0.3100ab | 0.2992ab | 0.2557b |

Note: The analysis was conducted among the top 15 genera with the highest bacterial abundance, and only the genera with significant differences were presented. The numbers represent the relative abundance of the genera, and different lowercase letters indicate significant differences (ANOVA LSD p<0.05).

**Table S3 Analysis of the differences in the relative abundance of dominant species of fungi at the phylum level**.

|  | 1YC | 2YC | 5YC | 10YC | 15YC | 20YC | 25YC |
| --- | --- | --- | --- | --- | --- | --- | --- |
| Zygomycota | 0.0817ab | 0.0681b | 0.0514b | 0.0667b | 0.1000ab | 0.0641b | 0.1325a |
| Glomeromycota | 0.0095c | 0.0428a | 0.0038c | 0.0219abc | 0.0112bc | 0.0364ab | 0.0112bc |

Note: The analysis was conducted among the top 15 phyla with the highest bacterial abundance, and only the phyla with significant differences were presented. The numbers represent the relative abundance of the phyla, and different lowercase letters indicate significant differences (ANOVA LSD p<0.05).

**Table S4 Analysis of the differences in the relative abundance of dominant species of fungi at the genus level**.

|  | 1YC | 2YC | 5YC | 10YC | 15YC | 20YC | 25YC |
| --- | --- | --- | --- | --- | --- | --- | --- |
| Mortierella | 0.0366ab | 0.0223b | 0.0225b | 0.0248b | 0.0364ab | 0.0279b | 0.0474a |
| Fusarium | 0.0125d | 0.0180cd | 0.0202cd | 0.0399a | 0.0285abc | 0.0251bcd | 0.0352ab |
| Ceratobasidium | 0.0003c | 0.0167bc | 0.0032c | 0.0054c | 0.0430ab | 0.0516a | 0.0145bc |
| Cortinarius | 0.0178a | 0.0138ab | 0.0058b | 0.0119ab | 0.0206a | 0.0144ab | 0.0133ab |
| Chaetomium | 0.0121ab | 0.0165a | 0.0089abc | 0.0066bc | 0.0084abc | 0.0087abc | 0.0023c |
| Glomus | 0.0048b | 0.0187a | 0.0017b | 0.0053b | 0.0051b | 0.0226a | 0.0043b |
| Thelebolus | 0.0018c | 0.0113ab | 0.0013c | 0.0154a | 0.0029bc | 0.0076abc | 0.0104abc |
| Cryptococcus | 0.0039b | 0.0026b | 0.0059b | 0.0034b | 0.0102a | 0.0032b | 0.0137a |
| Acremonium | 0.0271a | 0.0021b | 0.0043b | 0.0010b | 0.0007b | 0.0027b | 0.0006b |
| others | 0.1132ab | 0.1248a | 0.0661c | 0.0798bc | 0.0906ab | 0.1106ab | 0.1294a |

Note: The analysis was conducted among the top 15 genera with the highest bacterial abundance, and only the genera with significant differences were presented. The numbers represent the relative abundance of the genera, and different lowercase letters indicate significant differences (ANOVA LSD p<0.05).

**Table S5 Correlation analysis between continuous cropping years and soil physical and chemical properties.**

|  | CCY | SOC | TN | TP | C/N | C/P | N/P | TK | pH | RF |
| --- | --- | --- | --- | --- | --- | --- | --- | --- | --- | --- |
| CCY | 1 |  |  |  |  |  |  |  |  |  |
| SOC | -0.717* | 1 |  |  |  |  |  |  |  |  |
| TN | -0.717* | 0.992*** | 1 |  |  |  |  |  |  |  |
| TP | -0.618* | 0.908** | 0.891** | 1 |  |  |  |  |  |  |
| C/N | -0.655* | 0.934*** | 0.882** | 0.860** | 1 |  |  |  |  |  |
| C/P | -0.614* | 0.773* | 0.783* | 0.436 | 0.699* | 1 |  |  |  |  |
| N/P | -0.397 | 0.425 | 0.474 | 0.023 | 0.284 | 0.884** | 1 |  |  |  |
| TK | 0.087 | 0.477 | 0.476 | 0.658* | 0.441 | 0.035 | -0.24 | 1 |  |  |
| pH | 0.210 | -0.544 | -0.447 | -0.634* | -0.772* | -0.205 | 0.227 | -0.573 | 1 |  |
| RF | -0.147 | 0.189 | 0.07 | 0.172 | 0.492 | 0.119 | -0.164 | -0.158 | -0.638* | 1 |

Note: Pearson correlation analysis was adopted (n = 7). SOC, soil organic carbon; TN, soil total nitrogen; TP, soil total phosphorus ; C/N, carbon to nitrogen ratio; C/P, carbon to phosphorus ratio; N/P, nitrogen to phosphorus ratio; TK, total potassium; RF, residual film; CCY, continuous cropping years. *, ** and *** indicate significant levels of p<0.05, p<0.01, and p<0.001, respectively.


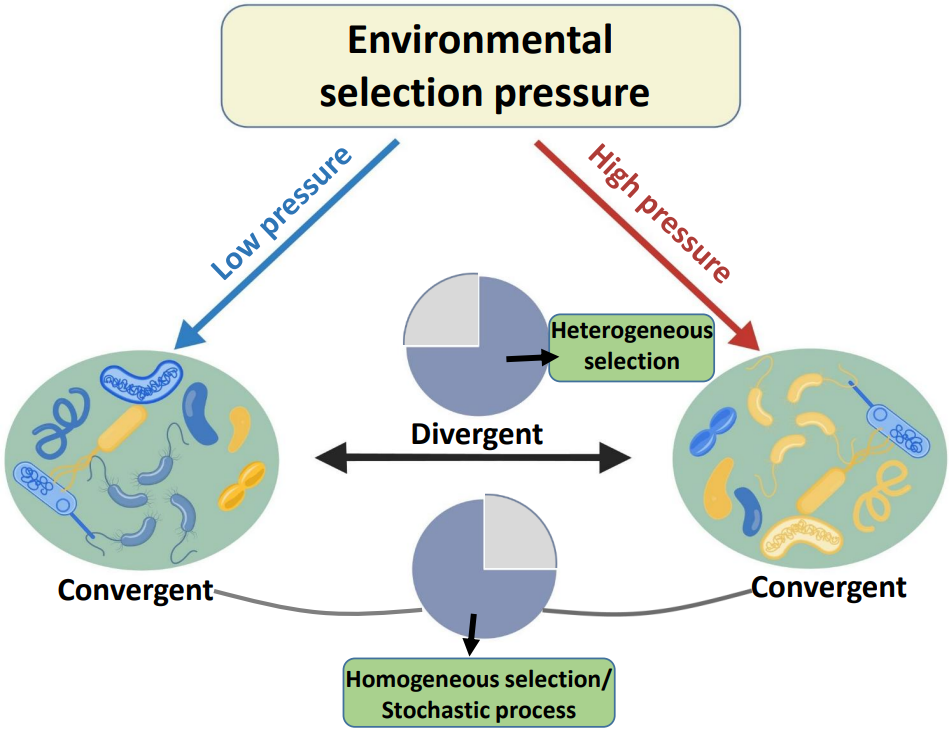


**Fig. S1 A schematic diagram of the convergent-divergent succession model.**


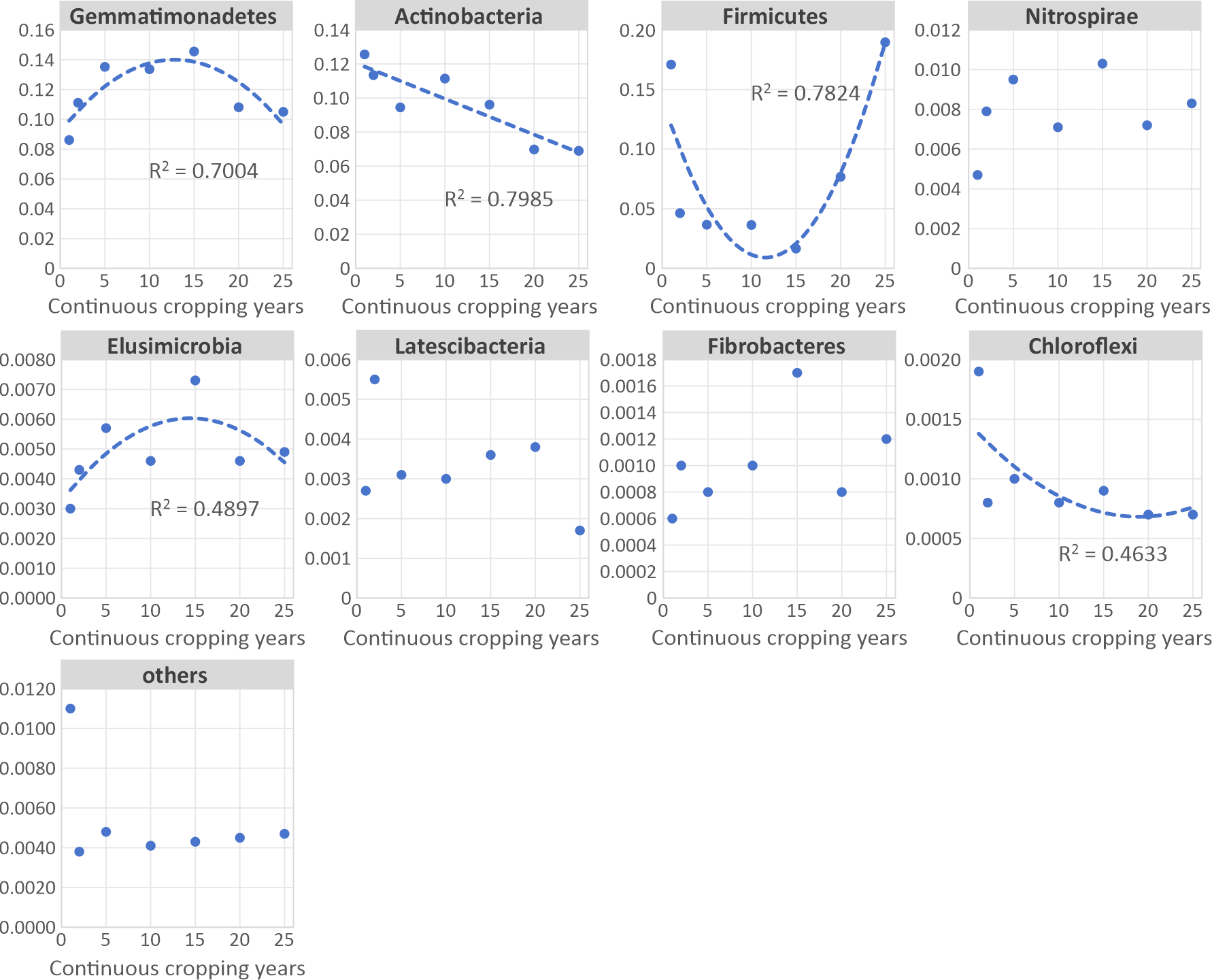


**Fig. S2 Regression analysis of relative abundances of bacterial taxa with significant differences at the phylum level against continuous cropping years.** The bacterial phyla shown correspond to Table S1. Linear regression was used when the goodness-of-fit was acceptable (R^2^≥0.3); otherwise, polynomial regression was attempted. Polynomial regression was deemed unacceptable if its R^2^<0.4.


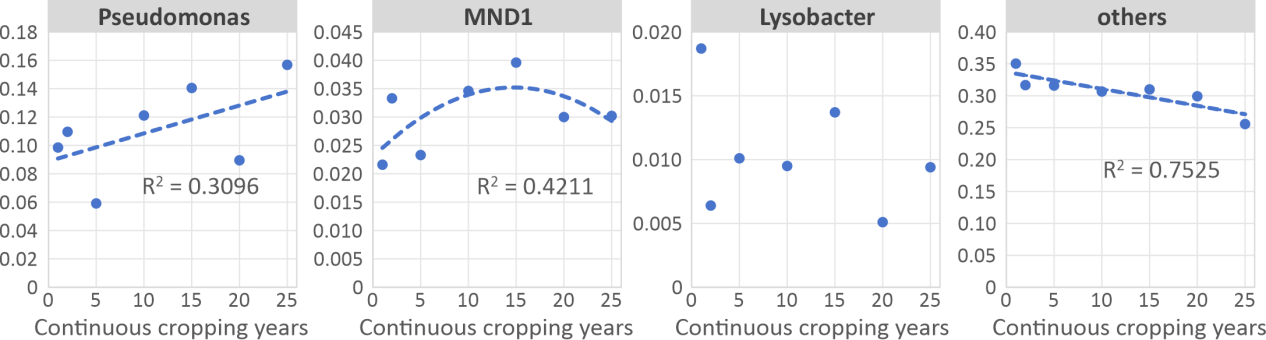


**Fig. S3 Regression analysis of relative abundances of bacterial taxa with significant differences at the genus level against continuous cropping years.** The bacterial genera shown correspond to Table S2. Linear regression was used when the goodness-of-fit was acceptable (R^2^≥0.3); otherwise, polynomial regression was attempted. Polynomial regression was deemed unacceptable if its R^2^<0.4.


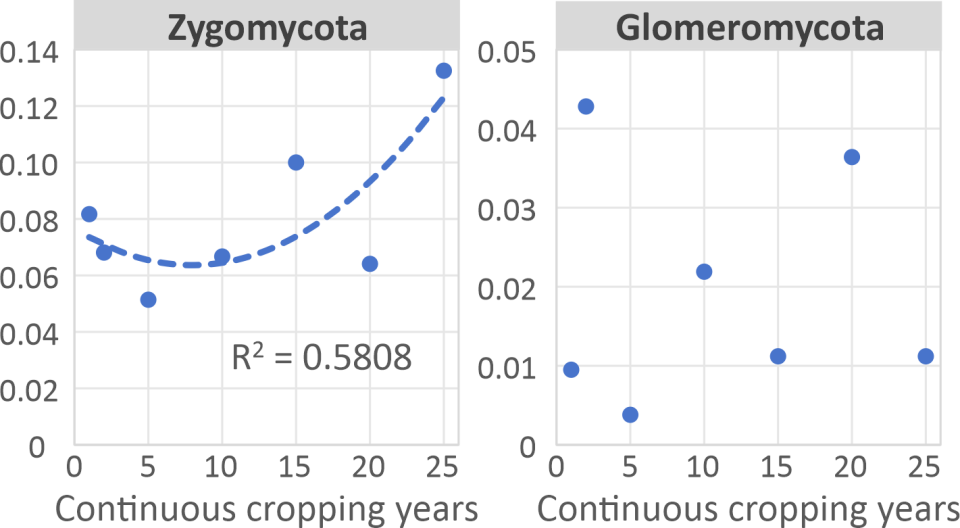


**Fig. S4 Regression analysis of relative abundances of fungi taxa with significant differences at the phylum level against continuous cropping years.** The fungi phyla shown correspond to Table S1. Linear regression was used when the goodness-of-fit was acceptable (R^2^≥0.3); otherwise, polynomial regression was attempted. Polynomial regression was deemed unacceptable if its R^2^<0.4.


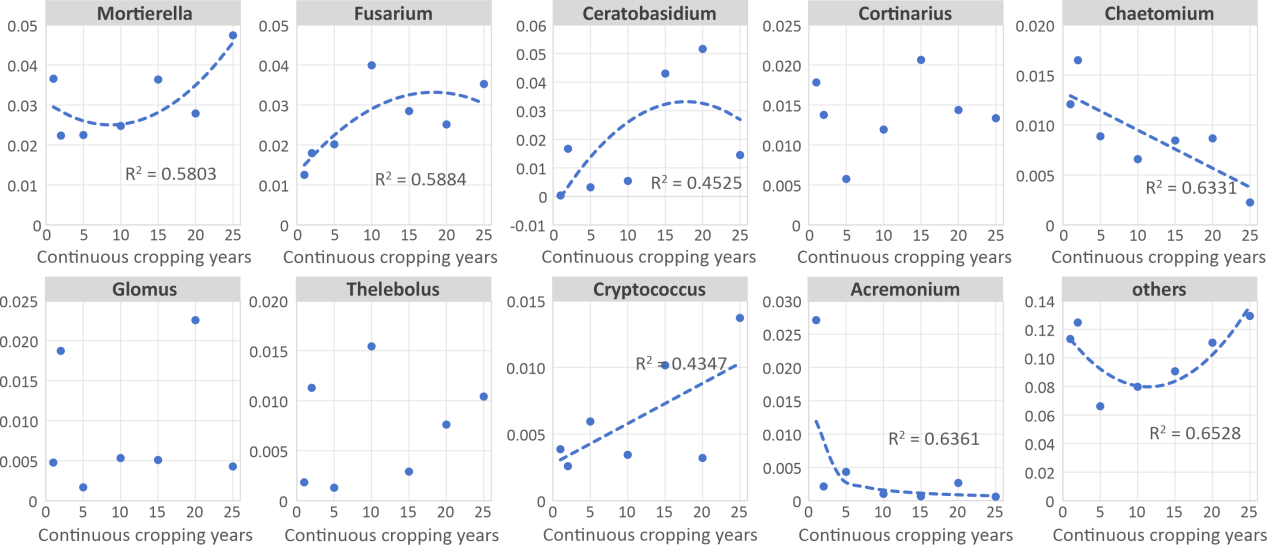


**Fig. S5 Regression analysis of relative abundances of fungi taxa with significant differences at the genus level against continuous cropping years.** The fungi genera shown correspond to Table S2. Linear regression was used when the goodness-of-fit was acceptable (R^2^≥0.3); otherwise, polynomial regression was attempted. Polynomial regression was deemed unacceptable if its R^2^<0.4.


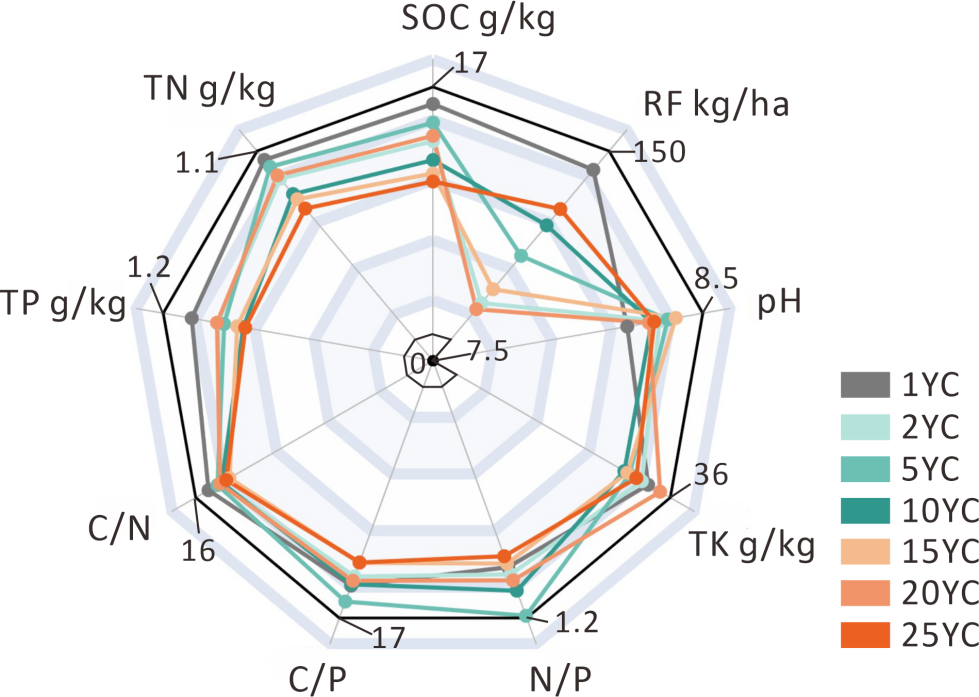


**Fig. S6 Radar chart visualization analysis of the impact of continuous cropping on soil physical and chemical properties.** SOC, soil organic carbon; TN, soil total nitrogen; TP, soil total phosphorus ; C/N, carbon to nitrogen ratio; C/P, carbon to phosphorus ratio; N/P, nitrogen to phosphorus ratio; TK, total potassium; RF, residual film. The range of the coordinate axes is indicated by the center origin to the outer black line. The center origin represents 0 for all except pH.

**Text S1 R code for network robustness analysis**

# Load required libraries

library(readr)

library(writexl)

library(ggplot2)

library(igraph)

library(dplyr)

# Define matrix data text (tab-separated values)

data_text <- "Pseudomonas uncultured_bacterium Ambiguous_taxa Escherichia-Shigella Sphingomonas MND1 Prevotella_9 Haliangium Dongia Lysobacter Bacteroides Acidibacter

Pseudomonas 0 0 0 0 0 0 0 0 0 0 0 0

uncultured_bacterium 0 0 0 0 0 0 0 0 0 0 0 0

Ambiguous_taxa 0 0 0 0 0 0.852472512071335 0 0.877992099174678 0.888362448303887 0 0 0.705364136656022

Escherichia-Shigella 0 0 0 0 0 0 0.997547764059729 0 0 0 0 0

Sphingomonas 0 0 0 0 0 0 0 0 0 0 0 0

MND1 0 0 0.852472512071335 0 0 0 0 0.929846495749121 0.930143236357863 0 0 0.766994542771665

Prevotella_9 0 0 0 0.997547764059729 0 0 0 0 0 0 0 0

Haliangium 0 0 0.877992099174678 0 0 0.929846495749121 0 0 0.928565082137789 0 0 0.711838324078005

Dongia 0 0 0.888362448303887 0 0 0.930143236357863 0 0.928565082137789 0 0 0 0.746984729161724

Lysobacter 0 0 0 0 0 0 0 0 0 0 0 0.890136692816334

Bacteroides 0 0 0 0 0 0 0 0 0 0 0 0

Acidibacter 0 0 0.705364136656022 0 0 0.766994542771665 0 0.711838324078005 0.746984729161724 0.890136692816334 0 0"

# Read data into a dataframe (first column as row names, first row as column names)

data_df <- read.table(text = data_text, row.names = 1, header = TRUE)

# Convert dataframe to matrix

adj_matrix <- as.matrix(data_df)

# Convert all non-zero values to 1 while keeping zeros intact

adj_matrix[adj_matrix != 0] <- 1

# Create a graph object from the adjacency matrix (undirected graph)

g <- graph.adjacency(adj_matrix, mode = "undirected")

# Calculate the number of nodes to remove (80% of total nodes)

total_nodes <- vcount(g)

num_nodes_to_remove <- ceiling(0.80 * total_nodes)

# Initialize results dataframe

result <- data.frame(

deleted_nodes = integer(),

remaining_nodes = integer(),

remaining_edges = integer(),

natural_connectivity = numeric(),

stringsAsFactors = FALSE

)

# Function to calculate natural connectivity of a graph

natural_connectivity <- function(graph) {

adj_matrix <- as_adjacency_matrix(graph)

eigenvalues <- eigen(adj_matrix)$values

N <- vcount(graph)

return(log(sum(exp(eigenvalues)) / N))

}

# Iteratively remove nodes with highest degree

for (k in 1:num_nodes_to_remove) {

# Identify node with highest degree

max_degree_node <- which.max(degree(g))

node_to_remove <- V(g)[max_degree_node]

# Remove the node from the graph

g <- delete_vertices(g, node_to_remove)

# Calculate remaining nodes and edges

remaining_nodes <- vcount(g)

remaining_edges <- ecount(g)

# Compute natural connectivity

nat_conn <- natural_connectivity(g)

# Append results to dataframe

result <- rbind(result, data.frame(

deleted_nodes = k,

remaining_nodes = remaining_nodes,

remaining_edges = remaining_edges,

natural_connectivity = nat_conn,

stringsAsFactors = FALSE

))

}

# Print results

print(result)

# Plot remaining edges vs deleted nodes

ggplot(result, aes(x = deleted_nodes, y = remaining_edges)) +

geom_line() +

geom_point() +

labs(

title = "Remaining Edges vs Deleted Nodes",

x = "Deleted Nodes",

y = "Remaining Edges"

) +

theme_minimal()

# Plot natural connectivity vs deleted nodes

ggplot(result, aes(x = deleted_nodes, y = natural_connectivity)) +

geom_line() +

geom_point() +

labs(

title = "Natural Connectivity vs Deleted Nodes",

x = "Deleted Nodes",

y = "Natural Connectivity"

) +

theme_minimal()

# Save results to Excel file

write_xlsx(result, "result.xlsx")

**Text S2 Analytical methods for soil physicochemical properties**

**Soil organic carbon**

The potassium dichromate oxidation-ferrous sulfate titration method was used. Air-dried soil samples were passed through a 0.149 mm sieve. Accurately weigh 0.5 g of the sample into a 500 mL hard glass test tube. Add 5 mL of 0.8 M potassium dichromate solution and 5 mL of concentrated sulfuric acid. After shaking well, insert the test tube into an iron wire basket and heat in an oil bath at 170–180°C for 5 minutes until the solution turns transparent light yellow-green. Using o-phenanthroline as an indicator, titrate with 0.2 M ferrous sulfate standard solution until the solution turns brick red as the endpoint.

**Soil total nitrogen**

The semi-micro Kjeldahl method was employed. Weigh 1.0 g of air-dried soil sample passed through a 0.149 mm sieve into a Kjeldahl flask. Add 1 g of potassium sulfate, 0.1 g of copper sulfate, and 3 mL of concentrated sulfuric acid. Gently shake, and after the vigorous reaction subsides, raise the temperature to 360–400°C until the digestion solution and soil mixture become grayish-white with a slight green tint. Continue digesting for an additional 30 minutes. After cooling, transfer the digestate to a semi-micro Kjeldahl distillation unit. For steam distillation: add 20 mL of 10 M NaOH solution to the distillation chamber, and absorb ammonia with 25 mL of 2% boric acid solution (containing 0.1% bromocresol green-methyl red mixed indicator). Stop distillation when the distillate volume reaches 50 mL, and titrate with 0.01 M sulfuric acid standard solution until the solution changes from blue-green to gray-red.

**Total phosphorus**

The sulfuric acid-perchloric acid digestion method was used. Weigh 0.5 g of air-dried soil sample passed through a 0.149 mm sieve into a 50 mL Teflon crucible. Add 10 mL of mixed acid (H₂SO₄:HCIO₄=3:2), heat on a hotplate, and continue digesting for 20 minutes after the solution starts to turn white. After cooling, dilute to 50 mL with deionized water and let stand overnight. The next day, take 5 mL of the clear supernatant into a 50 mL volumetric flask. Add 5 mL of 0.5 M sodium bicarbonate buffer (pH 8.5) and 5 mL of molybdenum-antimony-ascorbic acid color-developing reagent (prepared fresh by mixing 0.5 g ascorbic acid, 10 mL ammonium molybdate solution, and 3.3 mL potassium tartrate antimony solution). Dilute to volume, let stand in the dark for 30 minutes, and measure the absorbance at 700 nm. Calculate the total phosphorus content using a phosphorus standard curve.

**Total potassium**

The NaOH fusion-flame photometry method was applied. Weigh 0.2 g of air-dried soil sample passed through a 0.149 mm sieve into a nickel crucible. Add 1.5 g of analytical-grade NaOH, mix thoroughly with a glass rod, and compact the mixture. Heat in a muffle furnace at 400°C for 30 minutes, then raise the temperature to 700°C for 15 minutes of fusion until the melt becomes uniformly blue-green. After cooling, place the crucible in a 250 mL beaker, extract with 50 mL of boiling water, neutralize with 5% hydrochloric acid solution (v/v) until the melt is completely dissolved, transfer to a 100 mL volumetric flask, dilute to volume, and filter. The potassium content in the filtrate was determined using a flame photometer, with a standard curve prepared from potassium standard solutions.

**Soil pH**

The potentiometric method (soil-to-water ratio 1:5, w/v) was used. Weigh 10 g of air-dried soil sample passed through a 2 mm sieve into a 100 mL beaker. Add 50 mL of deionized water, stir for 2 minutes, and let stand for 30 minutes. Measure the pH of the suspension with a pH meter (completed within 1 hour), maintaining the temperature at 25±1°C during measurement.
